# Supplementary figures and images for: Coordination of cell envelope biology by Escherichia coli MarA protein potentiates intrinsic antibiotic resistance
Source: PLoS Genet. 2025 May 5;21(5):e1011639. doi: 10.1371/journal.pgen.1011639 (PMC12052159; doi:10.1371/journal.pgen.1011639)

**Figure S1**

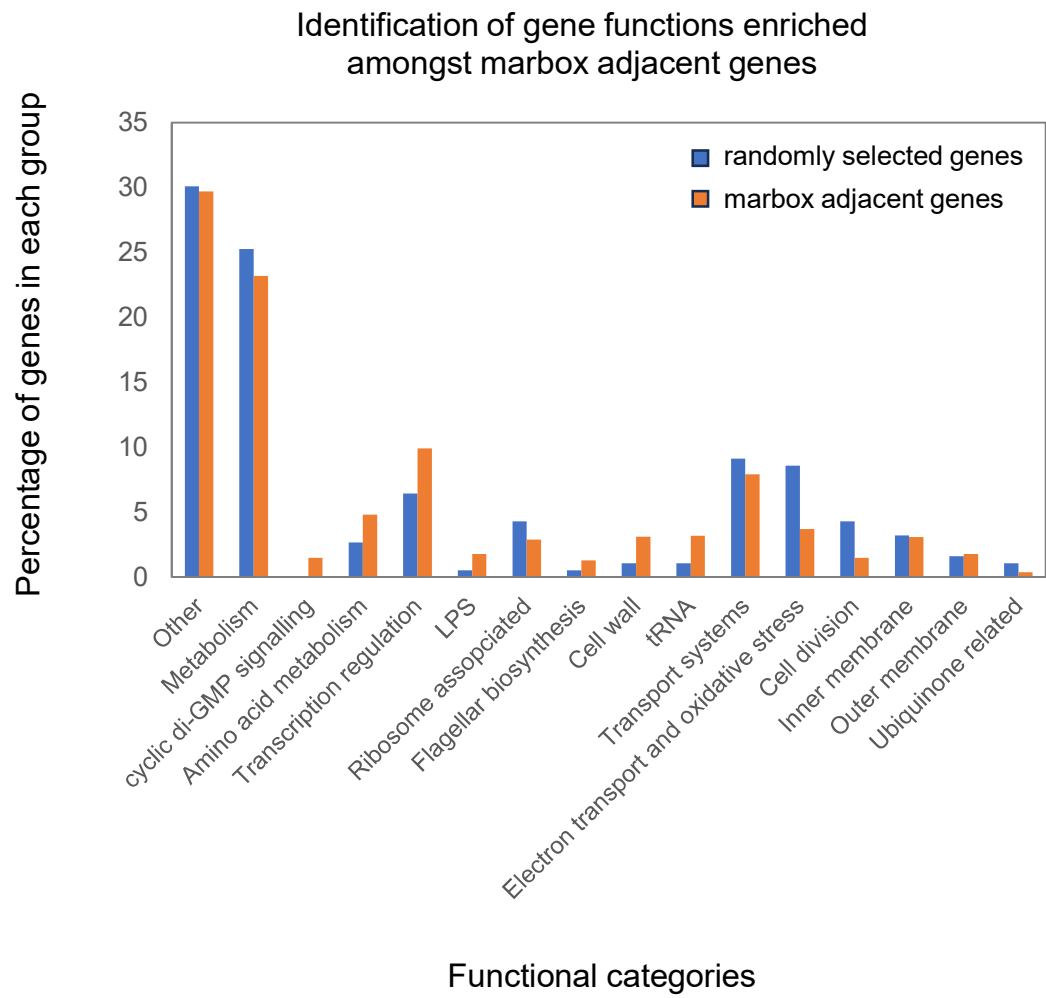

Supplement: S1 Fig — The bar chart shows the distribution of genes between different functional categories. Two groups of genes are represented: those selected randomly (blue) and those adjacent to a predicted marbox (orange). (PDF) [file pgen.1011639.s001.pdf]

**Figure S2**

**a**

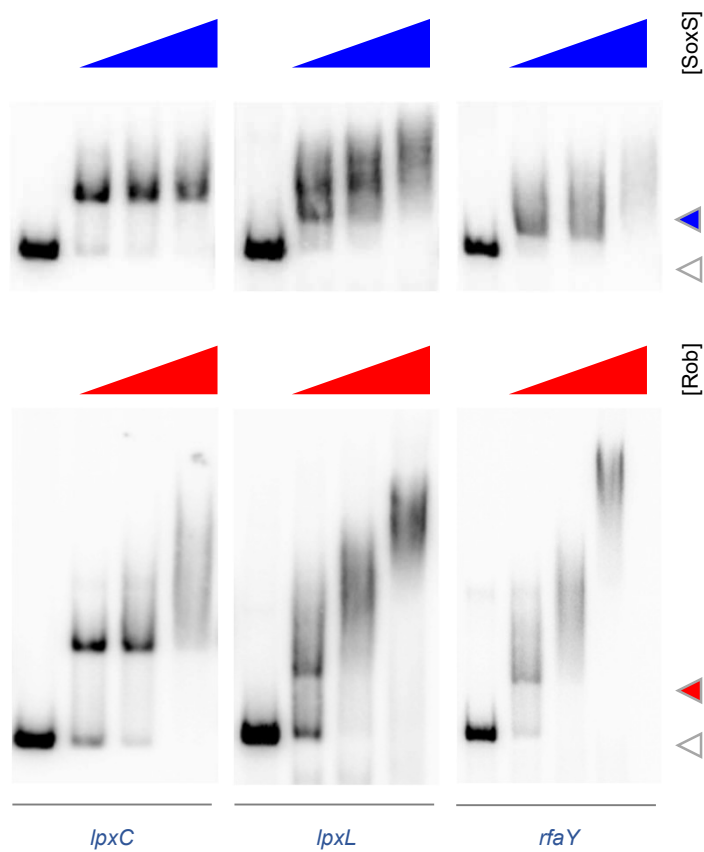

**b**

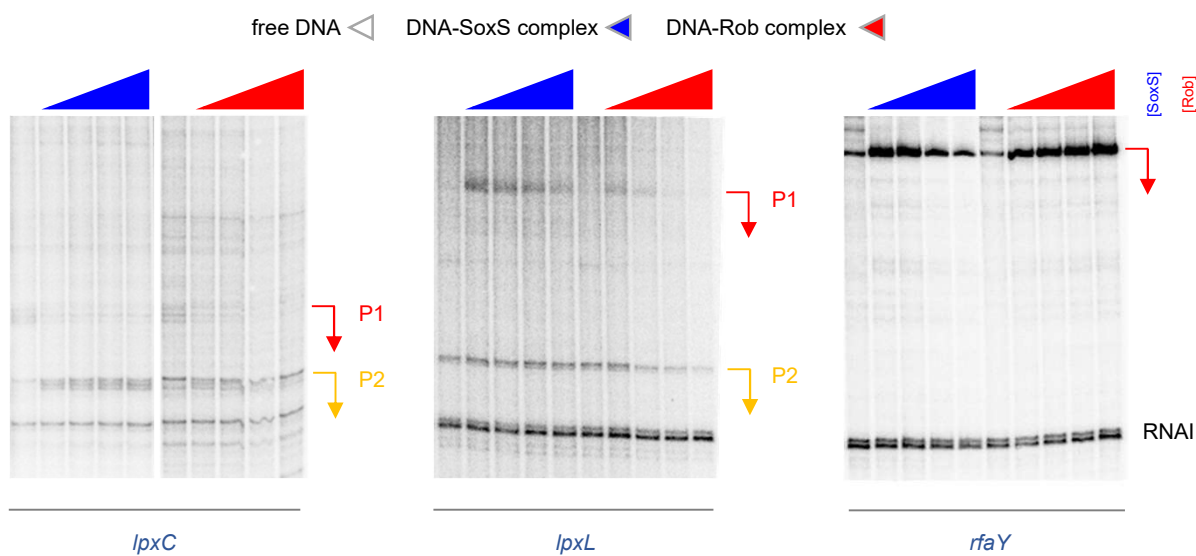

**c**

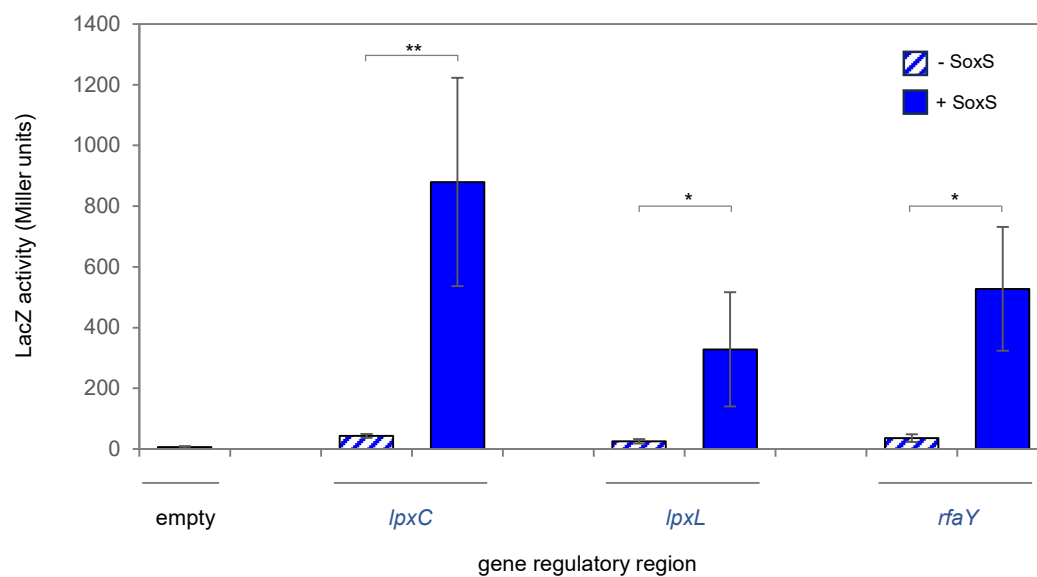

Supplement: S2 Fig — a. Binding of SoxS and Rob to the lpxC, lpxL, and rfaY regulatory regions. Results of electrophoretic mobility shift assays with the wild type derivatives of the three regulatory regions. Free DNA fragments are marked by an open triangle and SoxS-DNA or Rob-DNA complexes are highlighted by blue or red triangles respectively. Where present, SoxS was added at concentrations of 1, 2, or 4 μM. Conversely, Rob was used at concentrations of 0.2, 0.4 or 0.8 μM. Uncropped gel images are in S6 Fig. b. Control of lpxC, lpxL, and rfaY transcription by SoxS and Rob. The image shows results of in vitro transcription assays, using the indicated regulatory regions cloned in plasmid pSR upstream of the λoop terminator, as a DNA template. RNA polymerase σ70 holoenzyme was present at a concentration of 4 μM and SoxS was present at concentrations of 1, 2, or 4 μM. For Rob, concentrations of 0.2, 0.4 or 0.8 μM were used. The lpxC or lpxL P1 and P2 derived transcripts are indicated by red and orange bent arrows respectively. The rfaYP1 transcript is indicated by a bent red arrow. Uncropped gel images are in S6 Fig. c. Activation of lpxC, lpxL and rfaY transcription by SoxS in vivo. The bar chart shows results of a β-galactosidase activity assay using lysates from cells carrying the indicated regulatory regions, fused to lacZ in plasmid pRW50, in the presence (solid bars) or absence (striped bars) of ectopic SoxS production. Assays were done in triplicate on three separate occasions. Error bars show the standard deviation of the mean obtained from each set of triplicate experiments. Statistical significance was determined using a one-way ANOVA and post-hoc Tukey’s HSD test. Significant differences between groups are indicated where P is < 0.05 (*) or < 0.001 (**). (PDF) [file pgen.1011639.s002.pdf]

**Figure S3**

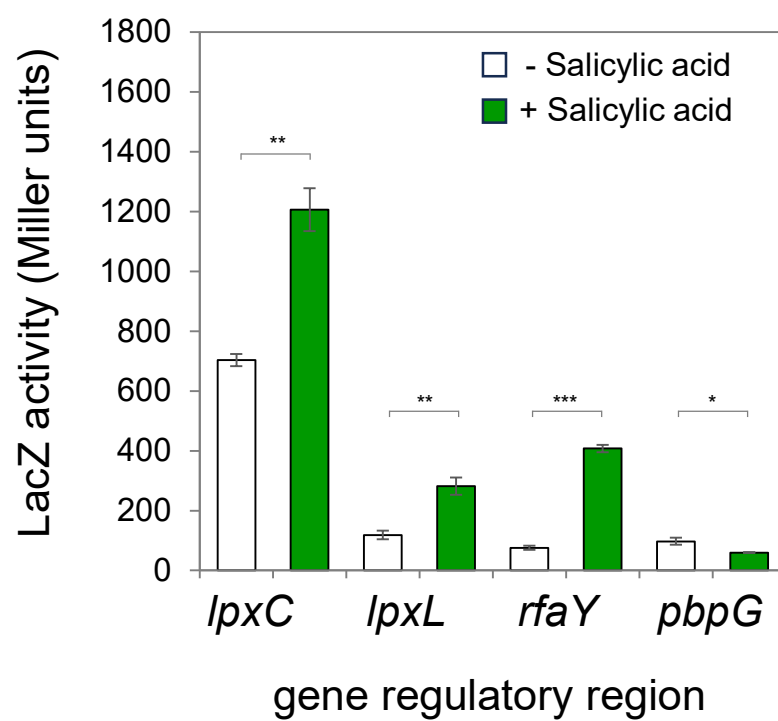

Supplement: S3 Fig — The bar chart shows results of a β-galactosidase activity assay using lysates from cells carrying the indicated regulatory regions, fused to lacZ in plasmid pRW50, in the absence (open bars) or presence (green bars) of 5 mM sodium salicylate. Error bars show the standard deviation of three biological repeat experiments. Statistical significance was determined using a student’s T-test. Significant differences between groups are indicated where P is < 0.05 (*), < 0.001 (**) or < 0.0001 (***). (PDF) [file pgen.1011639.s003.pdf]

Figure S4

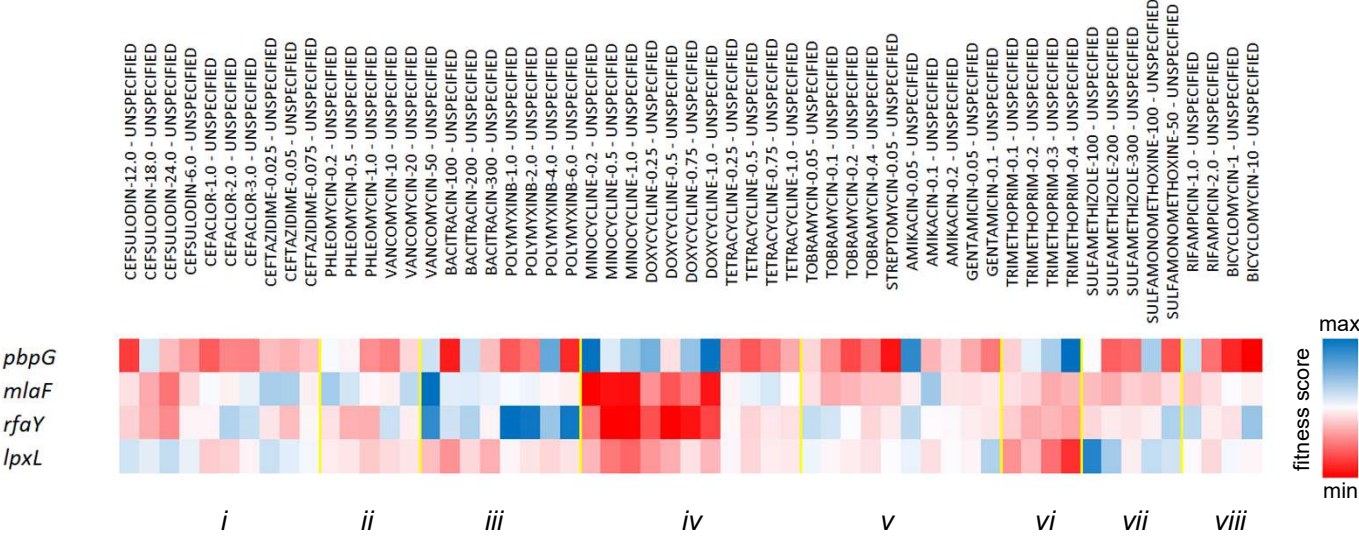

Supplement: S4 Fig — The figure shows a more detailed expansion of the heatmap in Figure 6b. (PDF) [file pgen.1011639.s004.pdf]

**Figure S5**

**a**

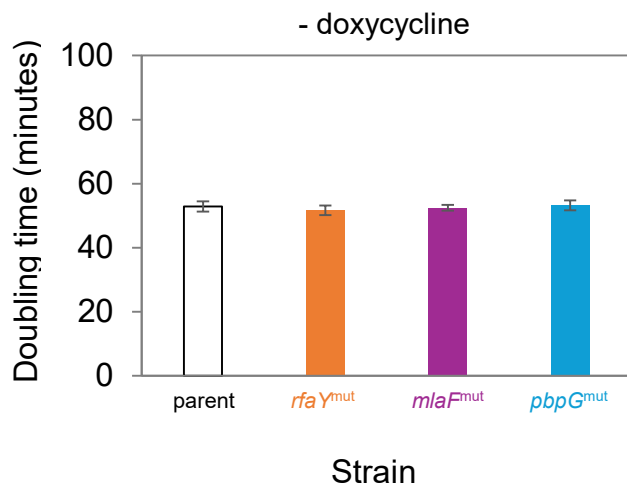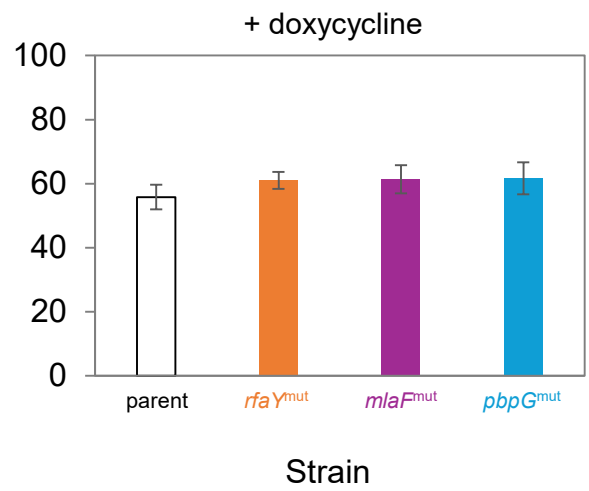

**b**

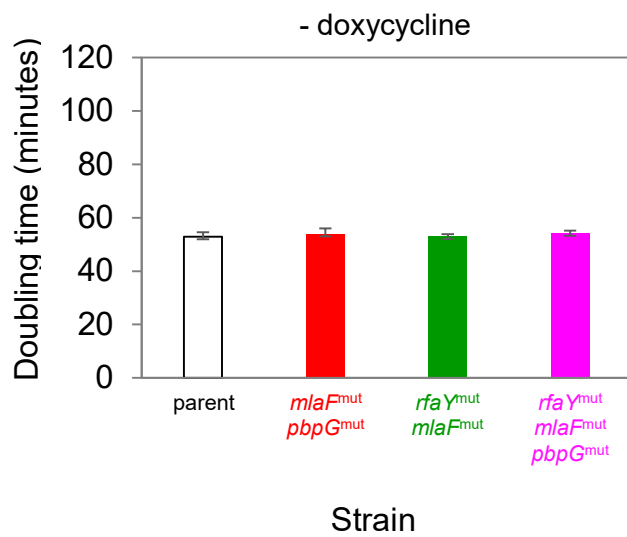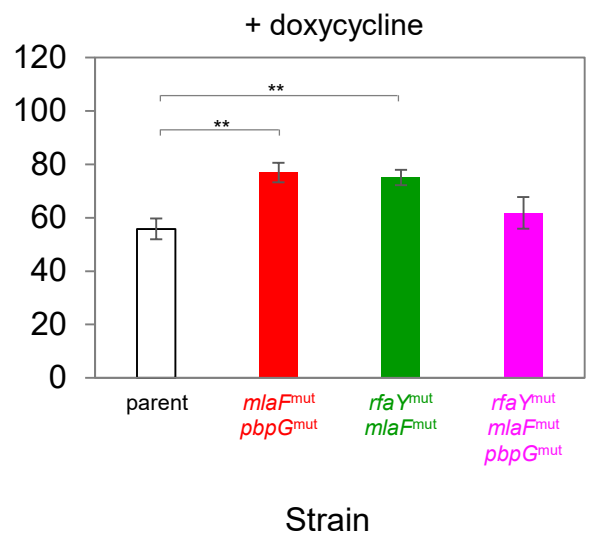

Supplement: S5 Fig — a. Doubling times for individual marbox mutations. The bar chart shows doubling times for strains carrying mutations in individual marboxes compared to the ΔthyA parent. Results are the average of three independent experiments and error bars indicate standard deviation. Statistical significance was determined using a two-tailed student’s t-test assuming unequal sample variance. b. Doubling times for combinations of marbox mutations. As for panel a, except that strains carry combinations of marbox mutations. Statistical significance was determined using a two-tailed student’s t-test assuming unequal sample variance. Where indicated (**) P = <0.01. (PDF) [file pgen.1011639.s005.pdf]

Figure S6

Figure 1c

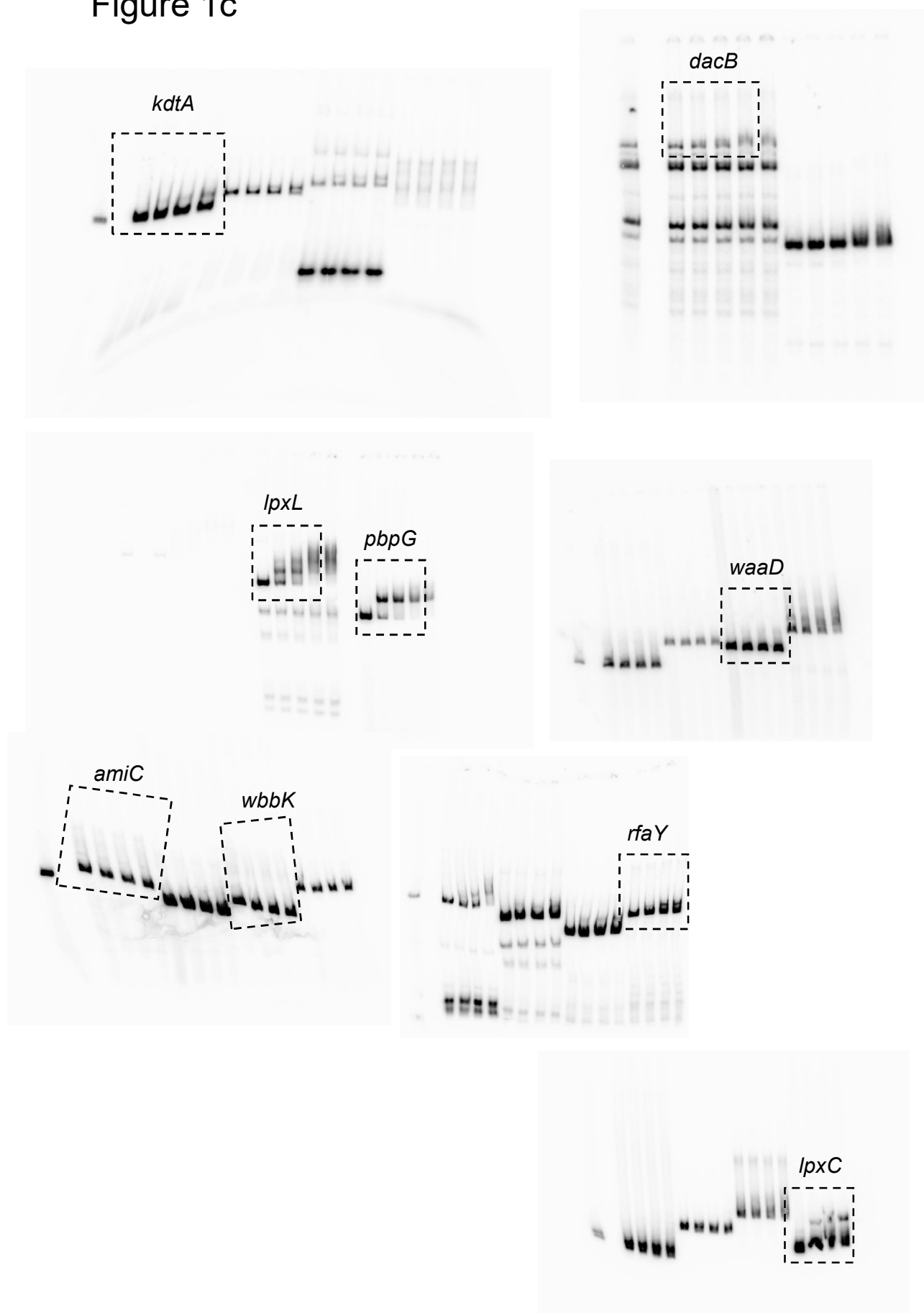

## Figure S6

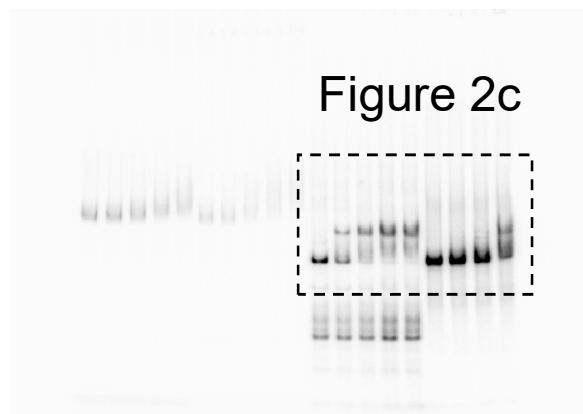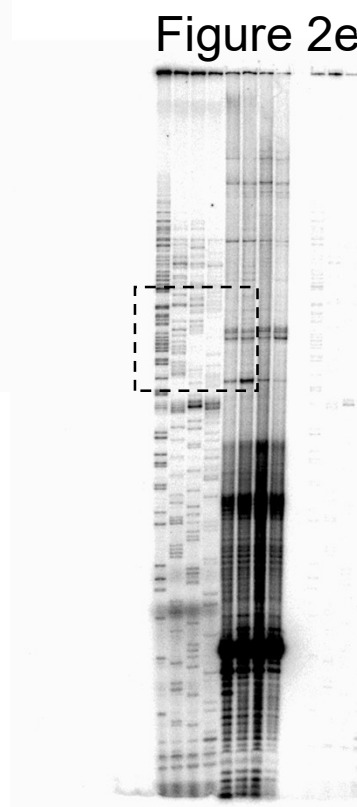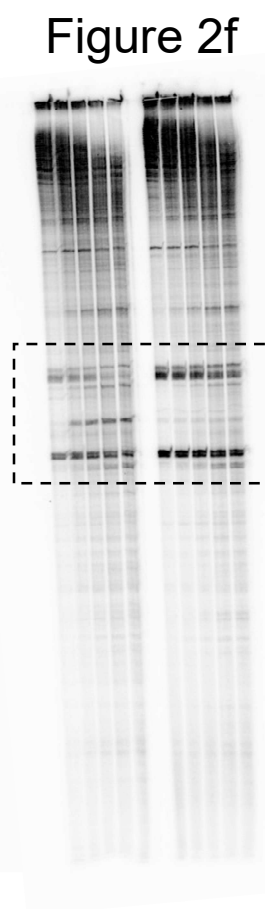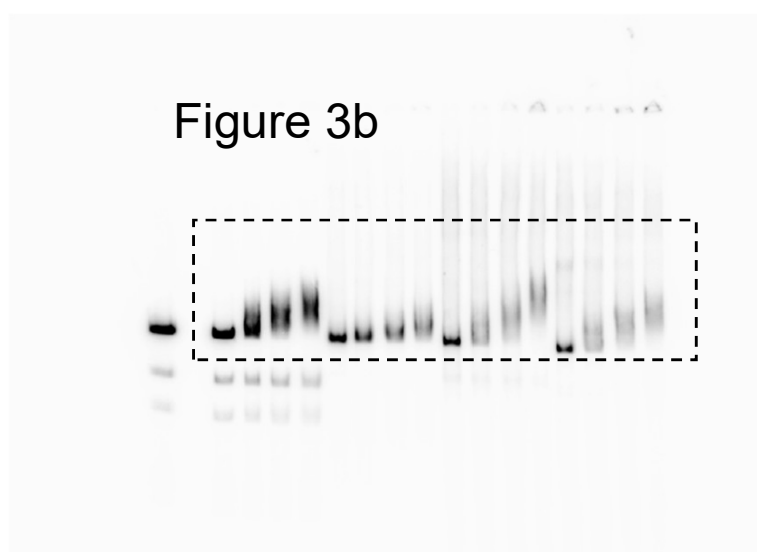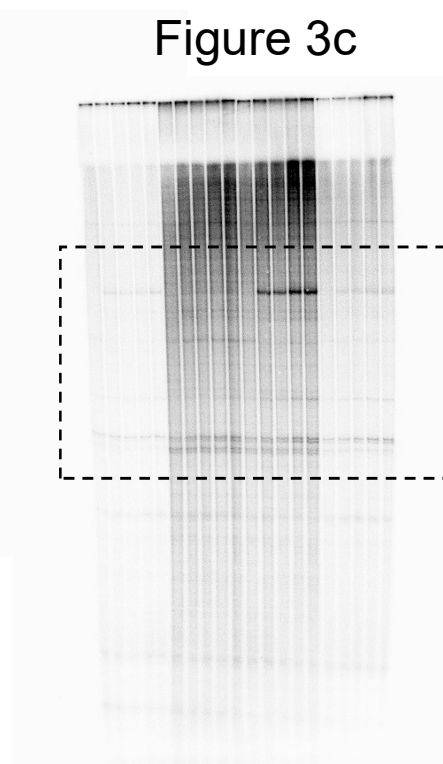

**Figure S6**

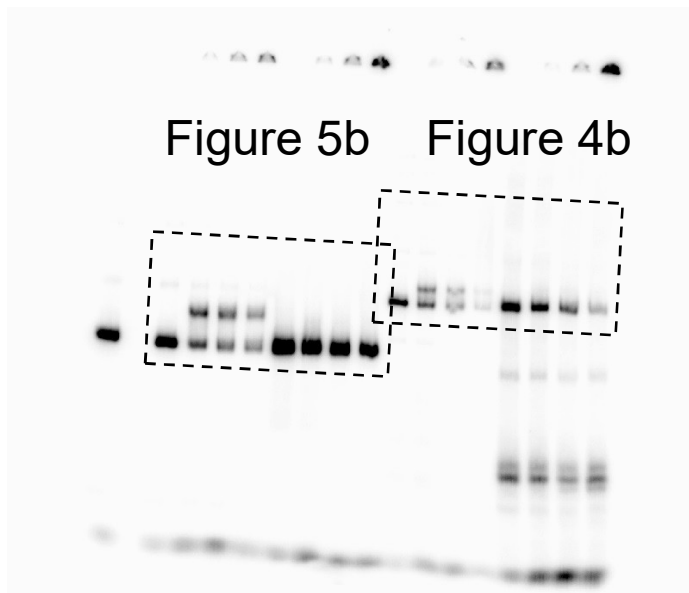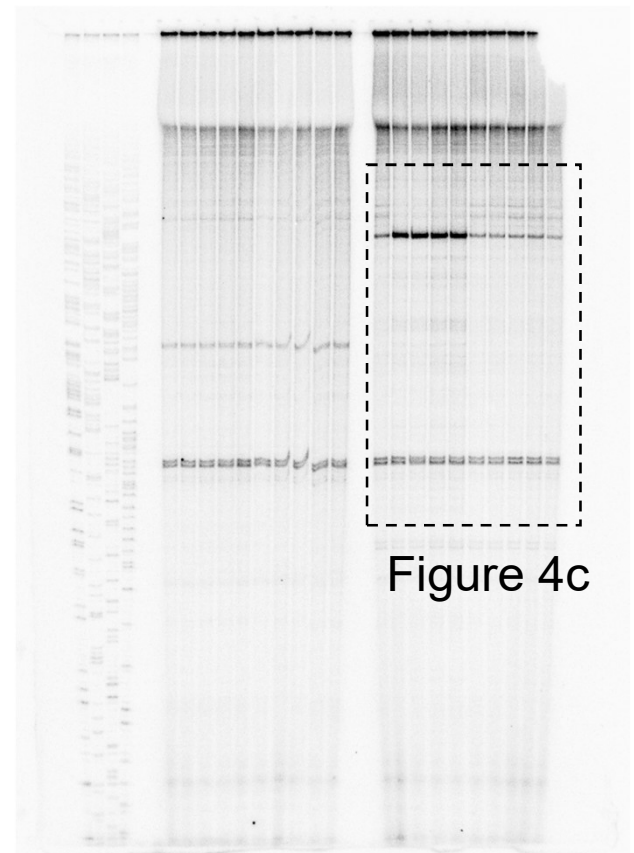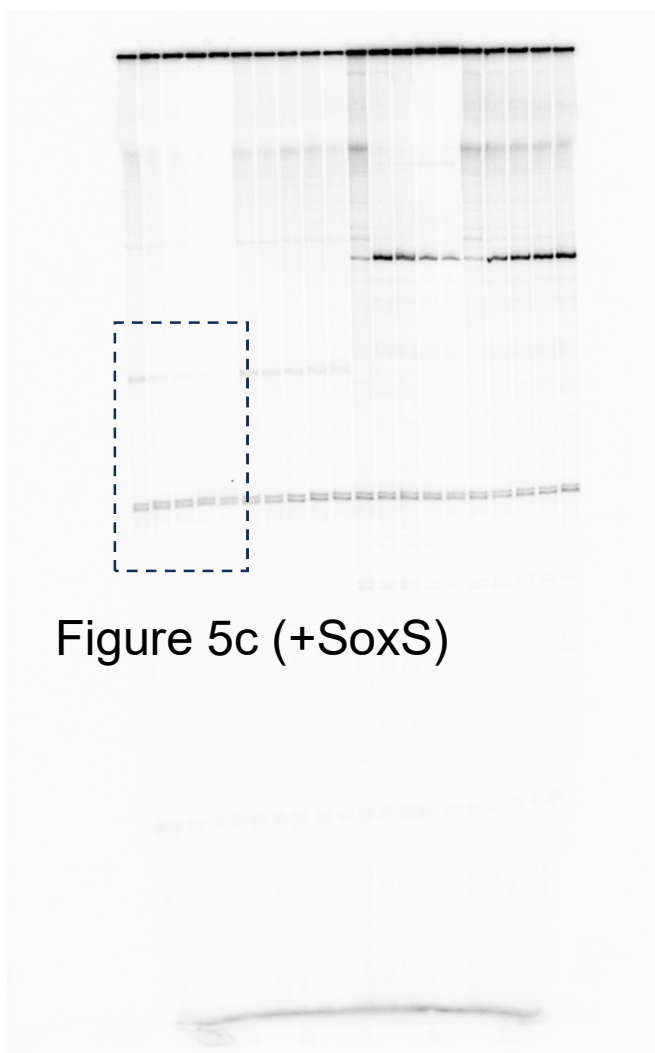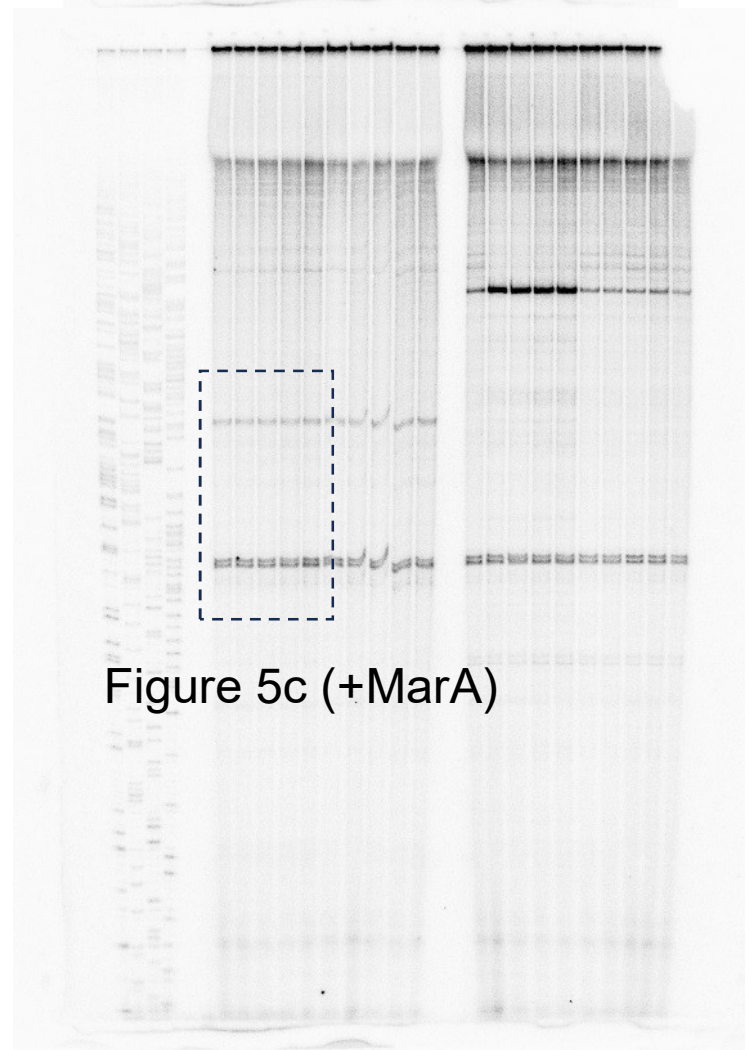

Figure S1a

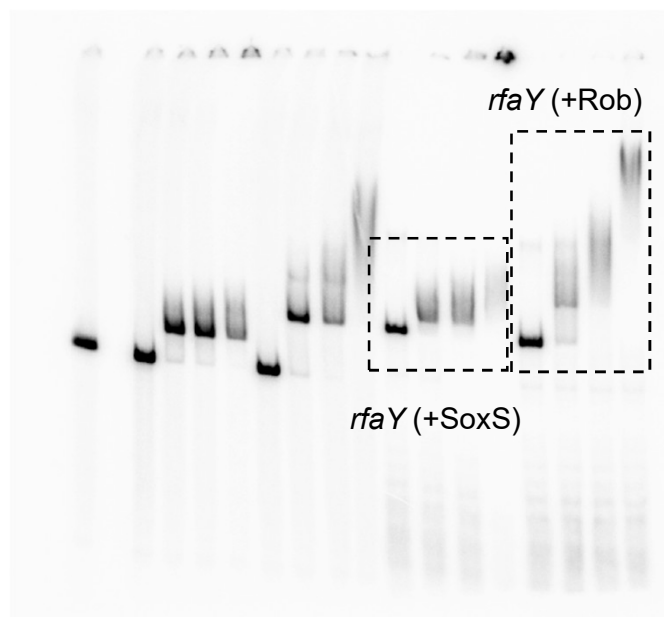

Figure S6

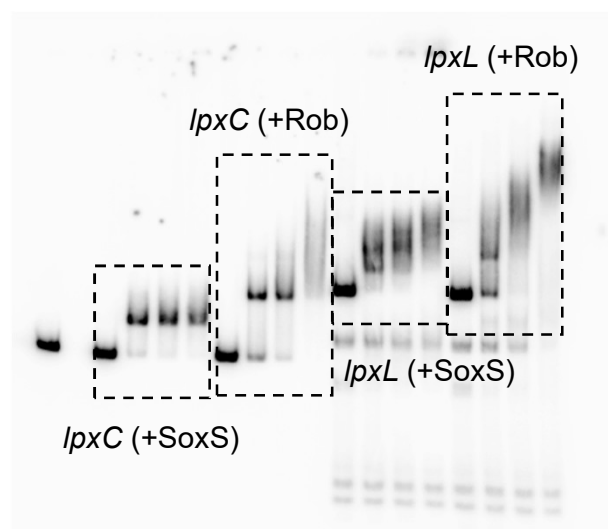

Figure S1b

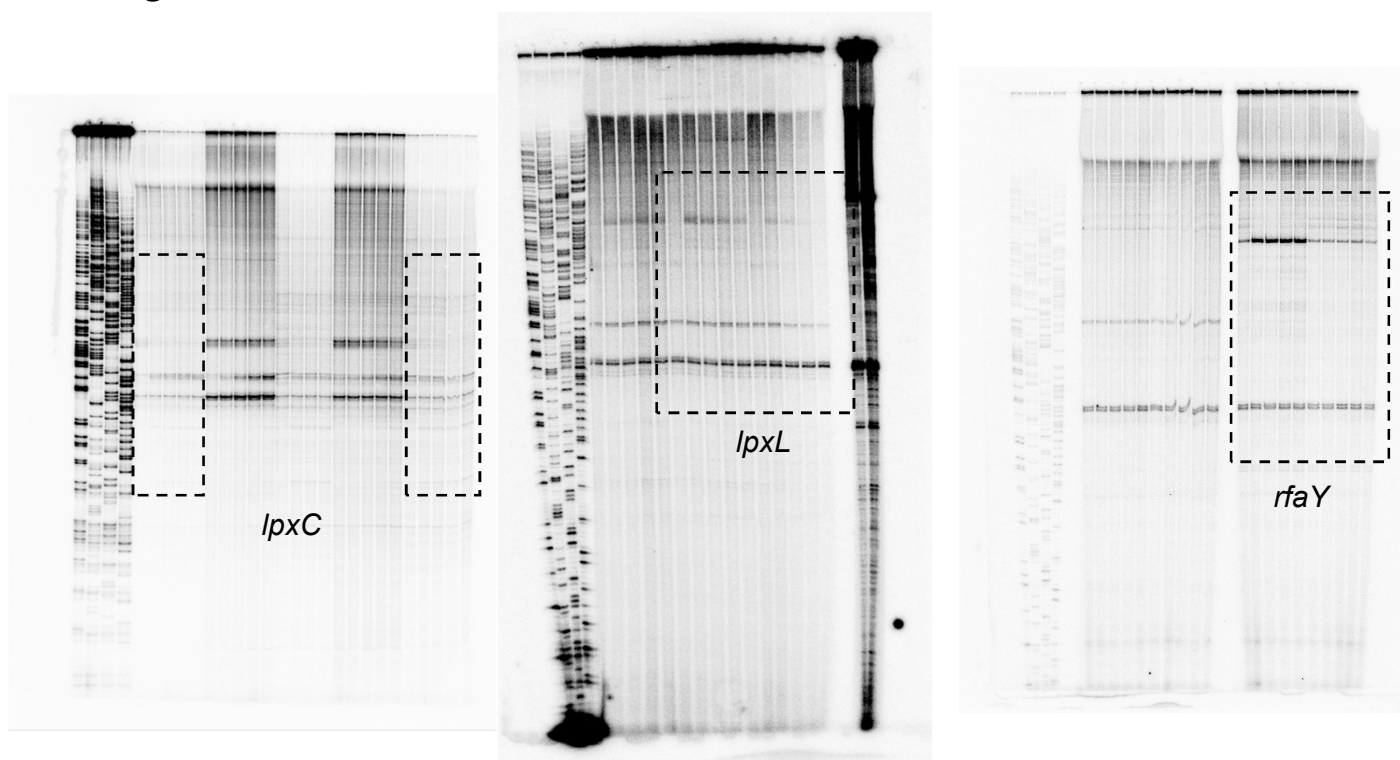

Supplement: S6 Fig — Regions of the gel images used in the main figures are boxed and labelled accordingly. (PDF) [file pgen.1011639.s006.pdf]
