# Supplementary material for: Coordination of cell envelope biology by Escherichia coli MarA protein potentiates intrinsic antibiotic resistance
Source: PLoS Genet. 2025 May 5;21(5):e1011639. doi: 10.1371/journal.pgen.1011639 (PMC12052159; doi:10.1371/journal.pgen.1011639)
Supplement: S1 Table — (DOCX) [file pgen.1011639.s007.docx]

Table S1: Strains, plasmids and oligonucleotides

**Name Description Source**

*Escherichia coli strains*

JCB387 Δ*nirB* Δ*lac* ^1^

JCB387 Δt*hyA* Δt*hyA* derivative of JCB387 This work

JCB387 Δ*thyA* *waaY*^mut^ Derivative of JCB387 Δt*hyA* with point mutations This work

GCA>TGT at positions -46 to -48 upstream of *waaY*P1

JCB387 Δ*thyA* *pbpG*^mut^ Derivative of JCB387 Δt*hyA* with point mutations This work

GCA>TGT at positions -25 to -27 upstream of *pbpG*P

JCB387 Δ*thyA* *mlaF*^mut^ Derivative of JCB387 Δt*hyA* with point mutations This work

CCA>TGT at positions -35 to -37 upstream of *mlaF*P1

JCB387 Δ*thyA* *pbpG*^mut^ Derivative of JCB387 Δ*thyA* *mlaF*^mut^ with point This work

*mlaF*^mut^ mutations GCA>TGT at positions -25 to -27 upstream

of *pbpG*P

JCB387 Δ*thyA* *waaY*^mut^ Derivative of JCB387 Δ*thyA* *mlaF*^mut^ with point This work

*mlaF*^mut^ mutations GCA>TGT at positions -46 to -48 upstream

of *waaY*P1

JCB387 Δ*thyA* *pbpG*^mut^ Derivative of JCB387 Δ*thyA* *waaY*^mut^ *mlaF*^mut^ with This work

*waaY*^mut^ *mlaF*^mut^ point mutations GCA>TGT at positions -25 to -27

upstream of *pbpG*P

T7 Express *fhuA*2 *lacZ*::T7 gene1 [*lon*] *ompT gal sulA*11 NEB

R(*mcr*73::miniTn10--T *etS*)2 [*dcm*]

R(*zgb*-210::Tn10--TetS) endA1 Δ(*mcrCmrr*)

114::IS10

*Plasmids*

pRW50 Broad-host-range lac fusion vector for cloning ^2^

promoters on *Eco*RI–*Hin*dIII fragments: contains

the RK2 origin of replication and encodes TcR

pSR pBR322-derived plasmid containing an *Eco*RI– ^3^

*Hin*dIII fragment upstream of the λ*oop* transcription

terminator

pET28a pET28a Protein expression vector with T7lac promoter Novagen

pJ203

pJ203*marA* pJ203 derivative encoding *marA* under the control of

a constitutive promoter.

pAM*soxS*

pACBSR Recombination plasmid containing arabinose inducible ^4^

λ red genes. Encodes CamR. Used for FRUIT.

pAMD001 pGEM-T containing the MG1655 *thyA* gene expressed ^5^

from a constitutive promoter. Encodes AmpR. Used for

FRUIT

*Oligonucleotides (sequence 5' to 3')*

lpxC.1-F GGCTGGAATTCTGCGTAAGCAAGCTGATTAAGAAT This work

lpxC.1M-F GGCTGGAATTCTGCGTAAGCAAGCTGATTAAGAATTG This work

ACTGGAATTTGGGTTTCGAGTGTGTTTGTGCTAAACT

GGCCCG

lpxC-R GCCCGAAGCTTCATCGTATTATCTCGCCAAATTACCT This work

AT

wbbK.1-F GGCTGGAATTCATTGGCCAGAGGGTTTGGTTG This work

wbbK.1-R GCCCGAAGCTTCATAATTTGGTCTCATGATTG This work

wzxC.1-F GGCTGGAATTCGCGCATAACGAACAGTATCG This work

wzxC.1-R GCCCGAAGCTTCATATCAATATGCCGCTTTGTTAAC This work

G

waaU.1-F GGCTGGAATTCGTAGGGATTTATTCAAAATATTG This work

waaU.1-R GCCCGAAGCTTCATTTTTATACCATATTATTTTAG This work

wcaD.1-F GGCTGGAATTCGGTGATTGCCACCCATAG This work

wcaD.1-R GCCCGAAGCTTCATACTCCTCCAGCATC This work

wzc.1-F GGCTGGAATTCATGCTGTTTGGTCACTGGG This work

wzc.1-R GCCCGAAGCTTCATTCTTATACCTGCTCTGCG This work

rfaJ.1-F GGCTGGAATTCGCAATCAATTAAGATATAGCGC This work

rfaJ.1-R GCCCGAAGCTTCACAATGCTACCCTTATATC This work

waaA.1-F GGCTGGAATTCGGGCCGAGTTTCAATGAATC This work

waaA.1-R GCCCGAAGCTTCATAGTAAATAGCTGACTTATGG This work

rffA.1-F GGCTGGAATTCGGACGCGGTGCAGGTG This work

rffA.1-R GCCCGAAGCTTCATGTGATCACCTGTATAACC This work

rfaD.1-F GGCTGGAATTCGCACTATTCACATGCAAAACCAAC This work

rfaD.1-R GCCCGAAGCTTCATAACTGTAACCTTCGAATTATG This work

waaY.1-F GGCTGGAATTCTGCAACTAAACCGTGG This work

waaY.1M-F GGCTGGAATTCTGCAACTAAACCGTGGCACAAATGGG This work

CAATTTATCCATCGGTAAAATACTATAAAATAGCTTT

AGAAAATTCCCCCTGGAAAGATGACTCTCCACGAGAT

CGGCCCTCAA

waaY.1-R GCCCGAAGCTTCATAATAAACCAGTTAAATG This work

lpxL.1-F GGCTGGAATTCCATGGTGTACGGTTCCTG This work

lpxL.1M1-F GGCTGGAATTCCATGGTGTACGGTTCCTGCGAGATGG This work

GAAAGTAAAAATCCGCGGCATGATATATGTATTATCG

ATAATTAACATCC

lpxL.1M2-F GGCTGGAATTCCATGGTGTACGGTTCCTGCGAGATGG This work

GAAAGTAAAAATCCGCGGCATGATATAGCAATTATCG

ATAATTAACATCCACACATTTTACGCTACATTACAGC

ATTAAAAATTATTTG

lpxL.1M3-R GCCCGAAGCTTCATATCAATCCTGTTTTTCAACCTAT This work

TCGGGCAATTGTATGTATTGTCGCATTTTTTCGCCCG

CAACCAAAATTTGTGGCTGAAGACTGGCGGCCCTTGC

CG

lpxL.1-R GCCCGAAGCTTCATATCAATCCTGTTTTTCAACC This work

amiC.1-F GGCTGGAATTCGTAAATTTTATGCGAGAGCGAC This work

amiC.1-R GCCCGAAGCTTCATGCCTCTCCCG This work

pbpG.1-F GGCTGGAATTCGGCGTAAATGTCAGCAATGC This work

pbpG.1M-F GGCTGGAATTCGGCGTAAATGTCAGCAATGCAGCATT This work

TCCTCACGATTCTCCTTTGACGATCTGTCTTTTTGCT

CGT

pbpG.1-R GCCCGAAGCTTCATGATGAGCATTCAGATAG This work

dacB.1-F GGCTGGAATTCCCTCTTGAATATTCCTGATGG This work

dacB.1-R GCCCGAAGCTTCATAATCTCGCGCTAACAAC This work

mepA.1-F GGCTGGAATTCGCATTACCGTGCCGG This work

mepA.1-R GCCCGAAGCTTCATTTTTTACCAGCGTGGAATATC This work

pSR-F CCATATATCAGGGTTATTGTCTC This work

pSR-R CATCACCGAAACGCGCGAGG This work

pRW50-F GTTCTCGCAAGGACGAGAATTTC This work

pRW50-R AATCTTCACGCTTGAGATAC This work

Universal primer GTAAAACGACGGCCAGT This work

D49724 GGTTGGACGCCCGGCATAGTTTTTCAGCAGGTCGTTG This work

pbpG-ORF-F GGCTGCATATGATGCCGAAATTTCGAGTTTC This work

pbpG-ORF-R GCCCGAAGCTTTTAATCGTTCTGTGCCGTCT This work

pbpG-flank-F GGCTGCTACGGTTATCGTTTGTTCTAG This work

pbpG-flank-R GCCCGGAACTGCTACAGCAG This work

lpxC-ORF-F GCCCGGAATTCTTATGCCAGTACAGCTGAAG This work

lpxC-ORF-R GGCTGGAATTCATGACGAATCTACCCAAGTTC This work

lpxL-ORF-F GCCCGAAGCTTTTAATAGCGTGAAGGAACGC This work

lpxL-ORF-R CGTCCTGAAATCACTCTGGTGACCA This work

lpxC-flank-F GATAATACAAAATATAATACAA This work

lpxC-flank-R GTGCGACGATAGATGACCCCGGTGT This work

lpxL-flank-F TACACTCGCCCAAAAACATTCAGCG This work

lpxL-flank-R ACGTTACTTGTCATCGTCATC This work

JW472 CCGACGCGCAGTTTA This work

JW473 CACGTTGTGTTTTCATGC This work

Targeting upstream waaY AAGACAAAACACATCAAAACTATAAAAAGCTGATTAC This work

AGAAAGTACTCTTCTTATTCATTATACAGGTGCAACT

AAACCGTAGACAGCTGCATGCAT

Targeting downstream waaY GGAGAGTCATCTTTCCAGGGGGAATTTTCTAAAGCTA This work

TTTTATAGTATTTTACCGATGGATAAATTGCCCATTT

GTGCCAGTGTAGGCTGGAGCTG

Mutagenesis upstream waaY GATGACATTATTTTTGCCTCGTGAG This work

Mutagenesis downstream waaY GCTATAATTCCTGAGATATAATGATGTTGCAC This work

Targeting upstream pbpG GTTCTAGAAAGTTCTTTGACGTTGCATTGCTGGCGTA This work AATGTCAGCAATGCAGCATTTCCTCACGATTCTCCTT

TGACGATAGACAGCTGCATGCAT

Targeting downstream pbpG TTCACGCGCACGGGTTGCGCACCGCCGGAGTAAGGAT This work

TTACTGAGGCTAGCGACGCCATCATAACGAGCAAAAA

GTGCGAGTGTAGGCTGGAGCTG

Mutagenesis upstream pbpG CTTAGCTTCTCTAGTTCGACGCTGG This work

Mutagenesis downstream pbpG CTGCGGTGCAAAAGGCACAGCCAGCA This work

Targeting upstream mlaF TTTCTTCAGGTATACTCGCCGGTCCGCTGAAGATTTT This work

CAGAAAGCCGTAACGGATGCTTAATTTTGACTTTATG

CGGCTATAGACAGCTGCATGCATCTTTGTTATGGTGT

GTTC

Targeting downstream mlaF CTCAATTTAACCTTGAACCCAACATATTTACAGAATA This work

TTACCCGCCGTGGTTAGCGAAAGCTGGCATTTGTTTT

ACTTTTGTGTAGGCTGGAGCTGTTAGATAGCCACCGG

CGCTT

Mutagenesis upstream mlaF CTTAGCTTCTCTAGTTCGACGCTGG This work

Mutagenesis downstream mlaF CGCATATCGACTAAATTCGC This work

Targeting upstream lpxC TGCGCCGCAAACTGCGAAAGAGCCGGATTATCTGGAT This work

ATCCCAGCATTCCTGCGTAAGCAAGCTGATTAAGAAT

TGACTGTAGACAGCTGCATGCAT

Targeting downstream lpxC CGCCAAATTACCTATCCAACCGAAGTGTACTATACAT This work

TCGGCGGGCCAGTTTAGCACAAAGAGCCTCGAAACCC

AAATTCGTGTAGGCTGGAGCTG

Targeting downstream TCGCCTGAACGATACGTTTAAGTGTCCTTTGTTTGAT This work

lpxC-promoter catCGTATTATCTCGCCAAATTACCTATCCAACCGAA

TTATAGCACAGATGCTGGATCTGTGTAGGTGTAGGCT

GGAGCTG

Targeting upstream lpxL CGCGTCGTTGGAAATGCGGTTGTGTAACACTGGCATG This work

GTGTACGGTTCCTGCGAGATGGGAAAGTAAAAATCCG

CGGCATTAGACAGCTGCATGCAT

Targeting downstream lpxL GCTTGTAAATAACAAATAATTTTTAATGCGCAAATGT This work

AGCGTAAAATGTGTGGATGTTAATTATCGATAATTGC

TATATCGTGTAGGCTGGAGCTG

Targeting downstream TGCGGTGGAGAACTTGGGTAGATTCGTcatATCAATC This work

lpxL-promoter CTGTTTTTCAACCTATTCGGGCAATTGTATGTATTAT

TATAGCACAGATGCTGGATCTGTGTAGGTGTAGGCTG

GAGCT

waaY M* GATGACATTATTTTTGCCTCGTGAGTACAATACAATT This work TATACAATTAAAAGTGAATTAAAAGACAAAACACATC

AAAACTATAAAAAGCTGATTACAGAAAGTACTCTTCT

TATTCATTATACAGGTGCAACTAAACCGTGTGTAAAT

GGGCAATTTATCCATCGGTAAAATACTATAAAATAGC

TTTAGAAAATTCCCCCTGGAAAGATGACTCTCCACGA

GATGCGAAATCAATTATTGAATTTAAAAAAAGATATA

AACATCTTTTAGTGCAACATCATTATATCTCAGGAAT

TATAGC

pbpG M* CTTAGCTTCTCTAGTTCGACGCTGGCGATTGGCGTGC This work TGGTACTGGAAGTGCTGCTCGCCCTAACCGTTATACT ATGGGGCTACGGTTATCGTTTGTTCTAGAAAGTTCTT

TGACGTTGCATTGCTGGCGTAAATGTCAGCAATGCAG CATTTCCTCACGATTCTCCTTTGACGATCTGTCTTTT

TGCTCGTTATGATGGCGTCGCTAGCCTCAGTAAATCC TTACTCCGGCGGTGCGCAACCCGTGCGCGTGAACCAC

TATCTGAATGCTCATCATGCCGAAATTTCGAGTTTCT

TTATTTAGCCTGGCCCTGATGCTGGCTGTGCCTTTTG

CACCGCAG

mlaF M* CAGGCGGTCGGCACTGTAAACGACCAAAAGTAAACCA This work

ACAATTAACAGTGCCGTAGCTAAAAGCATCTAACGTC CTTTCTTCAGGTATACTCGCCGGTCCGCTGAAGATTT TCAGAAAGCCGTAACGGATGCTTAATTTTGACTTTAT GCGGCTAAAAAGTAAAACAAATGTGTGCTTTCGCTAA CCACGGCGGGTAATATTCTGTAAATATGTTGGGTTCA AGGTTAAATTGAGCGCCATGCTTAGAAAATCAACGCA AGACGAAGGGTGAATTATGGAGCAGTCTGTGGCGAAT TTAGTCGATATGCG

waaY-amplify-F GGCTGCTAACAGAAAAAGCGTTGTC  This work

waaY-amplify-R GCCCGGATCTTGCTCTTCTGAATCAT This work

pbpG-amplify-F TGGTATTCCGAGCTTCCTGAATATC This work

pbpG-amplify-R CGCAACGGCCTGCGGTGCAAAAGGC This work

mlaF-amplify-F ATCAGCGGCGGGATGCCAAAGGTTC This work

mlaF-amplify-R CATCGCGCATATCGACTAAATTCGC This work

lpxC-amplify-F CGTCCTGAAATCACTCTGGTGACCA This work

lpxC-amplify-R GTGCGACGATAGATGACCCCGGTGT This work

lpxL-amplify-F TACACTCGCCCAAAAACATTCAGCG This work

lpxL-amplify-R AGCAGTGCGGTGGAGAACTTGGGTA This work

1. Page L, Griffiths L, Cole JA. 1990. Different physiological roles of two independent pathways for nitrite reduction to ammonia by enteric bacteria. *Arch Microbiol* 154:349–354.

2. Lodge J, Fear J, Busby S, Gunasekaran P, Kamini NR. 1992. Broad host range plasmids carrying the *Escherichia coli* lactose and galactose operons. *FEMS Microbiol Lett* 95:271–276.

3. Kolb A, Kotlarz D, Kusano S, Ishihama A. 1995. Selectivity of the Escherichia coli RNA polymerase Eσ38 for overlapping promoters and ability to support CRP activation. *Nucleic Acids Res* 23:819–826.

4. Herring CD, Glasner JD, Blattner FR. 2003. Gene replacement without selection: Regulated suppression of amber mutations in Escherichia coli. *Gene* 311:153–163.

5. Stringer AM, Singh N, Yermakova A, Petrone BL, Amarasinghe JJ, Reyes-Diaz L, et al. 2012. FRUIT, a Scar-Free System for Targeted Chromosomal Mutagenesis, Epitope Tagging, and Promoter Replacement in Escherichia coli and Salmonella enterica. *PLoS One* 7.

.
