# Supplementary material for: Coordination of cell envelope biology by Escherichia coli MarA protein potentiates intrinsic antibiotic resistance
Source: PLoS Genet. 2025 May 5;21(5):e1011639. doi: 10.1371/journal.pgen.1011639 (PMC12052159; doi:10.1371/journal.pgen.1011639)
Supplement: S2 Table — (DOCX) [file pgen.1011639.s008.docx]

**Table S2: Predicted marbox sequence and position relative to the nearest transcription start site**

**TSS^1^ predicted marbox position gene marbox sequence^3^**

**Marbox^2^ relative to TSS**

***Amino acid transport and metabolism***

149+ 160+ 11 thrL GCACAGACAGATAAA

3729- 3722+ 17 thrC ACGAGTACTGGAAAA

83743- 83745- 8 leuL TTTTAGAGTTACTGG

83811- 83828- -17 leuL ATTAGCTAATTGTGC

209626+ 209647+ 21 ldcC GCAGGCTATGATTAA

849378+ 849393- 15 ybiF TTGAGCATTTGTTGA

849434+ 849423- -21 ybiF TTTTGACTAAAATGC

899835- 899837- 8 artJ TTTGGCCGTGTCTGC

902310+ 902318- 8 artI TTTTCCATATACACC

2422546+ 2422562- 16 hisP GGTTTCACATGCTGC

2424859- 2424865- -6 hisJ TTACACTTATCGTGC

2926125+ 2926145+ 20 sdaC GCGGTAAATGATTAA

3084085+ 3084073- -22 speA TTTTTCATAACATTA

3237811+ 3237808+ -13 ygjU CCATTAAGTTTCAAA

3948288- 3948285+ 13 ilvL GTACTATTTACAAAA

3948314+ 3948337+ 23 ilvL GCAAGAAAAGACAAA

3950208- 3950200+ 18 ilvM CCAGTAATTCAGAAA

3951416- 3951437+ -21 ilvD TCAATAAATACAAAA

3955887- 3955876+ 21 ilvC GCAAAAATTGCAATG

3955887- 3955903- -16 ilvY TATATCAATTTCCGC

3320289- 3320279- 20 leuU TTTCCCATTCGCTAC

3597733- 3597748- -15 livJ TATTTCGTTTTATGC

3597817- 3597833- -16 livJ TTTGACAGTTTATTA

3851039- 3851041- 8 ivbL TTTAACAATATTTAT

2947087- 2947098+ -11 argA GAAACTTTAGCGAAA

2947125+ 2947116+ -19 argA GCATAATCTGTCATC

2947198+ 2947207+ 9 argA GCACCCTGCGAAAAA

3980292+ 3980284+ -18 argX GCATAAACTGGAGGA

698445- 698452- -7 asnB TGTTTAAAAAAATGC

2057701- 2057703+ 8 asnU GCAGAAAGTGAAACC

3303968+ 3303968- -10 mtr TTTTACAGATTGTGT

1321132- 1321127- 15 trpL TTTACGTGAACTTGC

914241+ 914235- -16 ybjE TTAATTACAAACTGC

4338739+ 4338727- -22 adiA TTTGGCTGATACTGA

2405541+ 2405562+ 21 yfbQ GCGATAACAGCAAAA

847277- 847323- -46 glnH TTTTCGAGATGCGGC

418648- 418679+ -31 brnQ GCGGTAAATCGAAAA

209626+ 209591+ -45 ldcC GCAAAAGTTCTGAAA

209626+ 209582+ -54 ldcC GCGTAATTCGCAAAA

209626+ 209593+ -43 ldcC AAAAGTTCTGAAAAA

2735497+ 2735474+ -33 pheL GCAACATCGGTGAAA

601152+ 601136+ -26 pheP GCACTAAATGTTAAA

3597817- 3597842- -25 livJ TTATTAACAATCTGC

189621- 189606- 25 map TTTCGCCAAACGTGC

289570- 289613- -43 argF TTTCACAATTCTTTC

1619191- 1619227- -36 ydeD TTTAACAGTTGATTC

3263114- 3263150- -36 tdcC TTTTGAATATATTGG

4338739+ 4338682- -67 adiA TTTTAAATTATTTTC

***Transcription regulation***

479647- 479644- 13 hha TTCTGAAGATCCTGC

17007- 17007- 10 gefL CTTTTCTCTATCTGC

63296- 63285- 21 hepA TATATCTTTAATTGC

257777- 257776+ 11 crl TAAAAATTTGCTAAA

257793+ 257795+ -8 crl GCCAATTTGGTAAAA

316412- 316427- -15 ykgA TGTATCAAAAATAGC

328588- 328592- 6 betI TTTATCTATTAAAGC

347727+ 347719- -18 prpR TTTCATAATTGTTGC

507408+ 507430+ 22 ybaQ GAAGCAACAGTTAAA

531509- 531506- 13 ybbS TTTATCAGTATTTTT

570491+ 570504+ 13 ybcM GCATGTCCTCCTAAA

570642- 570654+ -12 ybcM CCAGTTTATGAGATA

636947- 636954- -7 ybdO TTTTTCTAAAATAGC

1073321+ 1073332+ 11 ycdC AAAATATCTGGTAAA

1384703- 1384697+ 16 tyrR CCAAATAGTGTCATA

1493181+ 1493178- -13 ydcI ATTAACAATTAAAGC

1493235+ 1493246- 11 ydcI TTTGTAAATTCTTCC

1723981+ 1723989+ 8 ydhM GCTGTTAATGTCCAA

1735674+ 1735695+ 21 purR TCAAGATCGGCCAAA

1777463- 1777479- -16 ydiP ATTGCCGCAAATTGC

1934614+ 1934609+ -15 yebK GTAATATCTGACAAA

2212862+ 2212852+ -20 yehV GCGTCTAAAGTTAAA

2247775- 2247783- -8 yeiE TGTGGCATTTTATTC

2253312+ 2253310+ -12 yeiL GCTAAATATGAGAAA

2404669+ 2404673- -6 lrhA ATTATCACTTACTGG

2499010- 2499003+ 17 ypdC TCATTTTGTGTCAAT

2660296+ 2660293- -13 yfhP TTTAACCTTCAGTGC

2713408- 2713399- 19 yfiE TTTTTTAAATGTTCC

2859278- 2859293- -15 ygbI TTCTACGCTATTTGC

3204301- 3204321- -20 ygiP TTTCGCAAAACATTG

3670379+ 3670365+ -24 yhjC GCATTAGATGTTAGA

3939377- 3939370- 17 yieP TTTTTCTTTTTATTC

3939449+ 3939466- 17 yieP TTGCTGAAAAAATGC

4122537- 4122550- -13 cytR TTTAGCAGATAGCGC

4465385+ 4465385- -10 treR TATCGCCATAGATGC

4465429+ 4465439- 10 treR TTTTTCACTTACCGG

4593838- 4593844- -6 yjjM TTAATCACATTGTGG

4633383- 4633392- -9 rob AATAGCATTTTTTGC

4633383- 4633401- -18 rob TTTTGCTGTTTTAGC

1102479+ 1102495- 16 csgD ACTGACAGATGTTGC

4258176- 4258198- -22 zur TTTTAAATAAATTGC

1617544- 1617537+ 17 marA GGACGAAGTGGCAAC

2021860+ 2021853+ -17 rcsA GCAAATTATACAACA

4194859- 4194857- 12 rsd GTATTCAAAAACTGC

1292234- 1292253- -19 hns TTCTTTATTTTGTGC

4221763- 4221780- -17 iclR TTTTACCACAGATGC

3534641- 3534649+ -8 greB GCGTGTATTCTTAAA

3276701- 3276704- 7 agaR TTTCGTAAAACTTTC

1719001- 1718992- 19 slyA TTTCGCACTTAAAGA

1057207- 1057213- -6 torR CTGTTCATATTCTGC

852300+ 852307+ 7 ybiQ ATAGCTATTGCTAAA

3003869- 3003866- 13 ygeV GTTCACAGATCATGA

3003911- 3003926- -15 ygeV TTTATCATTTTCTAA

1289443+ 1289437+ -16 rssB GCCACTATTGAGTAA

2267943+ 2267954+ 11 spr GCATTTTTTTATAAC

1120377+ 1120365- -22 yceP TTTAGCATATTGTGC

877377+ 877371+ -16 yliH GTAAATTTTGTTAAT

1397576- 1397583- -7 fnr TTTGTCAATTTTAAC

2288386+ 2288387+ -9 narP GAAAATGATGCCAAA

2661372+ 2661381+ 9 suhB GCATCTTATCATAAA

1250245+ 1250268+ 23 ycgU ACACCTTTTGTCATA

3732847- 3732859+ -12 xylR GCAGTATATCGTTAA

3053843+ 3053838+ -15 ssrS GCAAGAAAAAGCAAA

3598893- 3598903- -10 rpoH TTTATCAGACCGTGA

3598950- 3598954- 6 rpoH TTACGCACAAAATGT

2288372+ 2288357+ -25 narP GAAGCTGATGCAAAA

17053- 17081- -28 gefL TTTAACCCATTTTGT

2165732+ 2165709- -33 ogrK TTATTCAGAAAATGT

877377+ 877362+ -25 yliH GAAGGAAATGTAAAT

877429+ 877412+ -27 yliH GCATTTTGCTCAAAA

1906924+ 1906902- -32 yobG TTCAGCGCATATTGC

3382625+ 3382608+ -27 argR ACGAAATTTGATAAA

3483974- 3483959+ 25 crp GAAAGCTATGCTAAA

3244629+ 3244613+ -26 exuR GCGAGTAGAGCTAAA

531509- 531559- -50 ybbS TTTGGAAAAATATTC

3204301- 3204332- -31 ygiP ATTGCCATTCCCTGC

829960- 829987- -27 ybiH CGTCACAAAAACTGC

4336044- 4336070- -26 adiY TTTTACTAATAGTAC

4336044- 4336089- -45 adiY TTTTATAGTAATTTC

1331785+ 1331769+ -26 cysB GCTATAAATGATATA

3065255- 3065292- -37 ygfI TTTTACAGAAAATTA

1934427- 1934492+ -65 yebK GGCAAAACTGATAAA

1934583+ 1934546+ -47 yebK TCATGTTATGAAAAA

1934427- 1934483+ -56 yebK GCAAAGTGCGGCAAA

3955887- 3955872- 25 ilvY CGTTGCAAAAATTGC

2696729+ 2696703+ -36 yfhH GCAATTAGCGGCAAT

1073321+ 1073282+ -49 ycdC GCCAGTTTTTAAAAA

4072668+ 4072651+ -27 yihW TCACTTTTTGTATAA

3372540- 3372592- -52 yhcK GTTTTCTCTTTGTGC

2937343+ 2937306+ -47 fucR GAATATTCTTTTAAA

1617117+ 1617142+ 25 marR GCAACTAATGTGAAA

1617117+ 1617054- -73 marC TTTAGCAAAACGTGG

3904597- 3904652- -55 bglG TATGTCAGGTTTTGC

1057207- 1057192- 25 torR TTTTACTCATTCTGT

2922642- 2922684- -42 csrB TTTTTAAGTATTCGC

2023546+ 2023486- -70 dsrA TTTGATAATTTGTGC

4047897+ 4047862+ -45 spf GCAACAACTGAAAAA

2680847+ 2680830+ -27 yphH GAACAATCTGGCAAT

529466+ 529418- -58 ylbG TTTTCCGCTTTTTTC

***Inner membrane proteins***

1653692+ 1653706- 14 ynfA TTTGTCTCTTATTGA

823823+ 823827+ -6 ybhQ GTAAGTAGAGATAAA

823678- 823691+ -13 ybhQ GCACCTTGAACGAAA

71184+ 71179+ -15 yabI GCGAGTATTTAAAAA

889233+ 889235+ -8 ybjM GCATGGAATGCGAAG

1099410- 1099407+ 13 ycdZ GAAGATTCTGAAGAA

1181022- 1181045- -23 ymfA CTTCATAAATGCTGC

1475621+ 1475616+ -15 ynbA GCACCATAGCGCAAA

1737851- 1737839+ 22 ydhC GCAAAATTTTTGAAA

1839485+ 1839485- -10 ynjF TATGGCATTTTTCGC

1839485+ 1839493- 8 ynjF TTTTCGCTTATCTGC

1852201+ 1852187- -24 ydjE TTTATCAGTTATTAT

2282215+ 2282217+ -8 yejM CAACGTACTGGAAAA

2282392- 2282379+ 23 yejM GCACTAAGGGAAACA

2671343+ 2671350+ 7 yphA ACATTATCTTGCAAA

2939759+ 2939757- -12 ygdD TTTACCCAGTTCTGC

3151432- 3151438+ -6 yghB GCGCGAATTGTATAA

3151453+ 3151457+ -6 yghB GCCACTTTTGGACAA

3245706- 3245716+ -10 yqjA GCAGAACATACCAAA

3247520+ 3247535+ 15 yqjE GCAGGCACTGAAACA

3338233+ 3338220+ -23 yrbG GCATAAAGTCAAAAT

3672782+ 3672796+ 14 yhjE CCAAAAAATAAGAAA

4001288- 4001296- -8 yigG TTTTTCAGAATATAT

4600928- 4600920- 18 yjjP TTGTTCAGATAATGA

4622834- 4622837- 7 smp TTTGAGACTTAATGT

2795107- 2795123- -16 yqaE TTTAGCGGAGCCTGC

3838118- 3838134- -16 nlpA TCTGGCACTTCTTAC

2960905- 2960923- -18 ppdC TCTGCCAGTAAACGC

2463194- 2463219+ -25 yfdC ACAGAAAAAGGCAAA

1843004+ 1842988- -26 ynjI TATTTCAGAATATGA

2898384- 2898460+ -76 yqcE TCAAATAATTTTAAA

***tRNA production***

214654+ 214648+ -16 yaeJ GCAAAATCAGAGCAA

1800596- 1800604- -8 thrS TATATCCTTATTTGC

2695939+ 2695945- 6 yfhC TATTCCAGTTGCTGG

2710924+ 2710939- 15 yfiC TTTTTCCGAACTTGA

2717083+ 2717092+ 9 yfiP TCATGATTTTCAAAA

2820157+ 2820181- 24 alaS TTTCGCCAAATCTCC

3208738+ 3208750- 12 ygjD TTTGAAATAAGCTGG

3511643+ 3511667- 24 trpS TTTTTCGATTCCTGA

4481950- 4481939- 21 valS TTTCAGAGAATCTTC

4481998+ 4481987- -21 valS GGTGGCAAAATCTGC

4390337+ 4390350+ 13 glyV GCAGAAATGCGAAAA

4390363+ 4390358+ -15 glyV GCGAAAATTACGAAA

1990179- 1990189- -10 glyW TCTGGCAATCAGTGC

696493- 696500- -7 metT TTCGTCAATTATTGT

3316348- 3316346- 12 metY TTGGGTAAAAAATGC

2945281- 2945292+ -11 metZ GCAAATTTTGCACAA

3108325+ 3108334+ 9 pheV GCAGTAAATTGAAGA

2519051+ 2519063+ 12 valX GCAAGAAATTGAGAA

2041628+ 2041626- -12 serU TTTAACAGTCCCTGT

2379604+ 2379610+ 6 elaC GAATATTTTTTGAAA

4173325- 4173333+ -8 thrU GCATAAAATGTGACC

3108325+ 3108298+ -37 pheV GCAATATCGGCAAAG

1262810- 1262843+ -33 hemA GCAATTACTCCAAAA

440600- 440635+ -35 thiI GCAAATAACGCGCAA

4397245+ 4397208+ -47 miaA GCAACTTGTGAAAAC

3408268+ 3408243+ -35 yhdG TCATCTCGTGCAAAA

3408268+ 3408252+ -26 yhdG GCAAAAAATGCGTAA

988267- 988314- -47 asnS ATTCGCAAATGCAGC

2518806- 2518832- -26 gltX GTTGACAAATTGCGC

3033188- 3033225- -37 lysS TTTCAAACTTGCTTC

2743402- 2743451- -49 trmD TTCTTGATAACCTGC

972627+ 972592+ -45 smtA GCAACATATTGCCAA

***Ribosome production***

1124690+ 1124693+ -7 rimJ GCAGAAAATGGCATT

1124745+ 1124734+ -21 rimJ GCAGGTATAGTAATA

1145934+ 1145936+ -8 yceD GCAATGTGTGCGAAT

1145945+ 1145946+ -9 yceD CGAATTATTGGCAAA

1145945+ 1145947+ -8 yceD GAATTATTGGCAAAA

1194231+ 1194253- 22 ymfC TTAACAAAAAATTGC

1496816+ 1496830+ 14 rimL GCATGAAAAAACAAA

1797938+ 1797927- -21 rplT TTGGTCAGAATGTGA

2734082+ 2734086- -6 rluD TTTAATATAGTGTGC

2744276- 2744292- -16 rpsP TTTACTCATTTTTGC

3208738+ 3208724+ -24 rpsU GCAATTGCAGTAAAA

3309772- 3309791- -19 rpsO TTAAACAATTTACGC

3331647+ 3331645- -12 rplU TTTTTCAGTACAATC

3442717- 3442734- -17 rplO TTTAATAGTCTTTGC

3444986- 3444976- 20 rpsN TTCGTCAGATTTCGC

3444986- 3444985- 11 rpsN TTTCGCAGTAGTGGT

3449904- 3449897- 17 rplW TCTTTCAGTTTCTGT

3472580- 3472597- -17 rpsL TTTAGCTTTTGCTTC

4605804+ 4605792- -22 rsmC ATTTGCTATATTTGC

4373634- 4373629+ 15 efp GCAAGTTGCGTTAAC

1007053+ 1007051+ -12 ycbY GCTCTTAGAGTAAAA

1264056- 1264074+ -18 prfA GCAGGATCTGGCATG

3034404- 3034407- 7 prfB TTGGCCAGATATTGT

189756- 189768+ -12 rpsB TCACTTTGTGTAACA

2753389+ 2753378+ -21 ssrA GCACGTATCATGAAA

2744276- 2744301- -25 rpsP TTTTGCGGGAATTGC

3208738+ 3208721+ -27 rpsU GCAGCAATTGCAGTA

3331538- 3331574- -36 rplU TTTGACAAGTGCTAC

3316027+ 3316007- -30 yhbC TCTGTTAATTTTTGC

***Flagellar biosynthesis***

1131025- 1131038+ -13 flgD GCATGATGCTGAAAA

1129377- 1129383- -6 flgM TTTATCATGTGTTGC

1129453+ 1129441- -22 flgM TTTATAAGAATATTC

1999851- 1999857- -6 fliA ATTATCCGTTTCTGC

2001649+ 2001643- -16 fliC GTTATCCTATATTGC

1243910+ 1243914- -6 ycgR GTAAGCATTTCTTGC

1130215+ 1130169- -56 flgA TTTTGCGTTTATTCC

1964323- 1964350- -27 flhB TTTGTCGGATGCGGC

2001699- 2001746- -47 fliC TTTTTCAAAAACAGC

2001699- 2001737- -38 fliC TTTAGAATTTTTTTC

2003203- 2003276+ -73 fliS GCAGCAGTTCGAAAA

2435894+ 2435853+ -51 flk GCATATTTTCATCAA

1790035- 1790076- -41 ydiV TTTTTCAGTATCCGG

***Ubiquinone related***

2337487- 2337510+ -23 ubiG GCAGTTTGAGGTAAA

3299339- 3299342+ 7 yhbU GCCATATCAGAAAAA

3299434- 3299422+ 22 yhbU GCCTTAAATCAAAAA

4016851+ 4016835+ -26 ubiE GCCCAATCTGTTACA

***Cell wall metabolism***

400172- 400172- 10 ddlA TCTAACACAAAGTGC

102171+ 102167+ -14 ddlB GGTAATATTGGAAAA

102171+ 102168+ -13 ddlB GTAATATTGGAAAAA

3520869+ 3520879+ 10 mrcA AAACTAAATGGGAAA

164652- 164649+ 13 mrcB GCACAAATTTTGAGA

164652- 164651+ 11 mrcB ACAAATTTTGAGAGA

2945281- 2945288- -7 mltA TGACGCAAATTTTGC

2823615- 2823616- 9 mltB TTTAACAGATTCAAC

233980- 233966- 24 mltD TGTGTCAATAGTTGC

1388717+ 1388732- 15 ycjI TTTGCCGGAAGTTGT

2061377- 2061400- -23 erfK TTTATCAGACTTTTC

980111+ 980119+ 8 ycbB TTAGGTTATGTAAAA

980232+ 980247+ 15 ycbB TCAGAGTATGATAAA

855092- 855081- 21 ybiS AATCACATTTTTTGC

855133+ 855147- 14 ybiS TATGGCATAATGCGC

778259- 778250+ 19 pal GCCGTATCTGTGATA

879769+ 879782+ 13 dacC GCAACAGCTGGCAAA

879841+ 879833+ -18 dacC GCATAAACTTTAAAA

2947087- 2947103- -16 amiC TTTAGCGAAAGACGC

1732405+ 1732408+ -7 ydhO TCAAGTTTTGTAAAT

2222963- 2222987+ -24 pbpG GCAAAAAGTGCGATC

2550192- 2550227+ -35 amiA CCAATATCTGACGAA

663338- 663372- -34 dacA GTTTGCAAACGTTGC

879841+ 879823+ -28 dacC GCAGGATATTGCATA

2080756+ 2080694- -72 dacD GTTGCCACTCGCTGC

233986- 234030- -44 mltD TTTATCAGAACCTAT

234114+ 234097- -27 mltD TTCTTCATTAATTAC

4628590- 4628648+ -58 slt GCAGTAAAAGTAAAA

2267832+ 2267815+ -27 spr GCACCAATCGGGTAA

1756888- 1756923- -35 ynhG TTTGGCGCGAATTGC

1169647- 1169700- -53 ycfS ATTTGTAAATGTTGC

***Cell division***

3600779+ 3600789- 10 ftsE TCGGGCAAAAAGTGC

4262283- 4262291+ -8 dnaB GCTTCATATGATAAA

1811729- 1811739- -10 cedA TTTAGCCGATTTAGC

1811848+ 1811848- -10 cedA TTTGACCAAAACAGC

1645949- 1645948- 11 dicC TTTAGCATTAGCTAA

3164048- 3164059- -11 parC TACTACATTATCTGC

4003726- 4003734+ -8 recQ GGGGGTTATGCTAAA

3958493- 3958508+ -15 rep GTAAACTCTGAAAAA

2870929- 2870948- -19 ygbQ TTTGTCCATTGTTAC

3929154- 3929151- 13 yieN TTCTGCGTAAATTGC

4048883+ 4048896- 13 yihA TTTGTGAGACATTGC

4325085+ 4325080+ -15 yjdA GCAGTAATTATAAAA

2529356- 2529390- -34 zipA TTTTGCTGTTTTTTC

1020306+ 1020284- -32 sulA TTAAGAATAAGTTGC

235991+ 235975+ -26 dnaQ GAACCATCGGTGAAA

***LPS production***

3806495- 3806481+ 24 kdtA GCTATTTAAGTCAAA

3806495- 3806491+ 14 kdtA TCAAAAACGGGAAAA

3806495- 3806502+ -7 kdtA AAAAGTAATGGTAAA

3794948- 3794939+ 19 rfaL GCAGTTTTGGAAAAG

3806261- 3806277- -16 rfaQ TGTATCATTTCTTTC

106508+ 106490+ -18 lpxC GCTCTTTGTGCTAAA

2493612+ 2493631+ 19 ddg GAATCTTCTGCGATA

1115961+ 1115972- 11 lpxL ATTATCGATAATTGC

1115961+ 1115982- 21 lpxL ATTGCTATATCATGC

1150692+ 1150688+ -14 acpP ATAATTAGTGCAAAA

1150764+ 1150764+ -10 acpP GGATTTAGTTGCAAA

1150773+ 1150774+ -9 acpP GCAAATTTTTCAACA

1938208+ 1938227- 19 msbB TTTTCCAGTTTCGGA

3799159- 3799200- -41 rfaY GCACAAATGGGCAAT

2363738+ 2363718- -30 ais ATTCGCAGTTGGTGT

2493515+ 2493499+ -26 ddg TCAGCGAGTGATAAA

***Outer membrane proteins***

2310789- 2310797- -8 ompC TATGCCTTTATTTGC

2310789- 2310807- -18 ompC TTTGCTTTTTTATGC

2310866- 2310866- 10 ompC TCTCCCCAAAAATGC

584967+ 584974- 7 ompT ATTATCACAATGCGC

4622181+ 4622184- -7 lplA TTTCTCGCTTTCTTC

220073- 220091- -18 rcsF TATTACAAATTGTGG

3175951+ 3175953+ -8 tolC GCACTAATAGTGAAT

3175951+ 3175964+ 13 tolC GAATTAAATGTGAAT

3176023+ 3176044+ 21 tolC GCACGTAACGCCAAC

3176084+ 3176070+ -24 tolC GCGGCTTCTGCTAGA

2032044- 2032034+ 20 yedS GCACTTATTGATATA

4022869- 4022879+ -10 yigC GCAAGGATAGCAAAA

1653667- 1653676+ -9 ynfB GCAAAAAATAGTAAC

3111645- 3111653- -8 yghG TTTTGTAATAACGGC

3079895- 3079901+ -6 yggG GCATTTTTAGAATAA

2751557+ 2751541+ -26 smpA GCAAAAGGTTTTAAA

1311899+ 1311874+ -35 yciD GAAAATATTTAAAAA

1262810- 1262839- -29 hemM TTCGGCAATTACTCC

***Transport systems***

4244564- 4244572- -8 malE TGTTACAGAATTGGC

47211+ 47211+ -10 yabF ACACTTTGTGGGAAA

77532+ 77534+ -8 yabM GCATCATGTGTGACT

289893+ 289896- -7 afuB ATGTTCAGATAATGC

418738+ 418730+ -18 brnQ GCTAAAATTGTTTAA

728096+ 728111- 15 kdpF TTCGCAAAAATCTGC

793024+ 793023- -11 modF TATCCCACTTTGTGC

945064+ 945067+ -7 ycaD GCATGAAATTTGAAC

2085283- 2085275- 18 yeeF TTAGTCATTTTTTGA

1599413+ 1599404+ -19 ego GCATAAATTGTGATC

2183815- 2183827+ -12 yohM GCAACACCTGGTTAA

2183841- 2183842+ 9 yohM CAAGAATATGAAAAA

2057988+ 2057990- -8 yeeO TTTTGCTATATCTGA

2058003+ 2058026- 23 yeeO CTTTCCATCACCTGC

1881175+ 1881177+ -8 yeaV GCAAATTTTGTTACG

1836567- 1836580+ -13 ynjD GCAGTATATGCCAAC

2510891+ 2510900- 9 yfeP ATTTACATAACTTTC

4328343+ 4328351+ 8 proP TCATTAACTGCCCAA

4592775+ 4592783- 8 yjjL TTTACGTAATTGTGC

3864367- 3864367- 10 yidE TTTGCCTTATAGCGC

3609866+ 3609862+ -14 yhhT ACACTTAATACAAAA

3027074+ 3027066- -18 ygfS GTTAACATATCGCGC

3372814- 3372822+ -8 yhcL GCAAAAAATATAACA

1271318+ 1271314+ -14 chaB ACACTTAATGTTCAA

4133991+ 4133998+ 7 yijE GCGGCATGGGTGAAA

1914208+ 1914210+ -8 yebS GCATTAAATGCGTTA

3338238- 3338252- -14 yrbF TTTCTGAAAATCTTC

2350578- 2350586- -8 glpT TTTCGAATTATGAGC

3538177+ 3538164+ -23 feoA CCAGTTAATGAGAAA

3150038- 3150033- 15 exbB AGTCACAAAATCTGC

4364804- 4364819- -15 dcuA TTTTTAACAAGTTGA

4364845- 4364847- 8 dcuA TTTTTAAGTTACTGC

3604445+ 3604446+ -9 zntA TCGGTTAATGAGAAA

3128209+ 3128210- 1 ynaI TTTGTCATCTGGAGC

3463062- 3463055+ 17 gspM GAAAAAACAGAAAAA

1308460- 1308455- 15 kch TTTTCCTGAATATGC

2723848+ 2723848- -10 kgtP TTATACATTTGTTGC

4019786+ 4019783+ -13 tatA GTATTAAGTGGCACA

4020150+ 4020146+ -14 tatB GATTTTACTGCGAAA

776924+ 776919+ -15 tolB TAACATTCTGCTAAA

1080461- 1080469+ -8 ycdN ACATCATTTTATAAA

3826819- 3826810+ 19 yicE GCTGTTAATGAACAA

1271151- 1271167- -16 chaA TTTACGAATTTTTAC

1817927- 1817935- -8 chbA AATCGCATTTTGTGC

2184763+ 2184757+ -16 yohN GCATAATGCGATAAC

611889- 611890+ 9 fes GCAAATAGTTATCAA

611929+ 611926+ -13 fes GCAATCAATGAAAAA

1156887- 1156900+ -13 ptsG ATATGTTTTGTCAAA

3103721- 3103722+ 9 nupG TCACGATGTGAGGAA

3025101- 3025107+ -6 ygfQ GCAACTAATAATAAA

4007108- 4007115- -7 yigK TGTGCCACAATACGC

3067915- 3067929- -14 yggB TTTGCCAAATAGATC

846491+ 846469- -32 glnP TTTTACAGGTGTAGC

617434+ 617417+ -27 fepE TCACCAACTGGTAAA

4246003+ 4245958+ -55 lamB ACAGGTGATGTGAAA

2310886- 2310921- -35 ompC TGTTTCATTTATCGC

3906351- 3906404- -53 pstB TTTTCCTCATCGTGC

1353648+ 1353631- -27 sapB ATGGGCAATTCCTGC

431380+ 431364- -26 tsx TTTCACAGTTCTATC

4240314- 4240356- -42 xylE ATTATCATTTTTTAC

445911- 445965- -54 yajR TTTAGTAAATATTTC

844930+ 844858- -82 ybiO TTTGCCGGATGCGGC

1045062+ 1045043- -29 yccZ TTTATCAGTCAATGT

1619289+ 1619262+ -37 ydeE GCATTAATGCGCAAA

1767109+ 1767083+ -36 ydiK ACAAAAAAGGTGAAA

2614048+ 2614073- 25 yfgO TTTCACAGTATCGGT

3119339+ 3119324- -25 yghK ATTATCTTTATATGC

3826944+ 3826899+ -55 yicE TGAGGATGTGGCAAA

3839806- 3839842- -36 yicM TTTTGCAACTTTTAC

4276504+ 4276469+ -45 yjcD GCATCTTGACGAAAA

4277939+ 4277914+ -35 yjcE GCACATTGTGTGAGC

1697338+ 1697311+ -37 malX CTAAAATTTGATAAA

1899857- 1899888+ -31 manX GAAATTTCTGCTAAT

3826831- 3826860- -29 gltS TATATAAAAAAGTGC

4419681- 4419713+ -32 sgaA GCAGGATGTGAAGTA

4387491- 4387527- -36 yjeP TTCGGTAGATACTGC

2666070+ 2666016- -64 yfhS TTGCAAAAATAATGC

1760764+ 1760715- -59 ynhD TTCACCAATACGTGA

2228625+ 2228599+ -36 yohJ GCACGTTTTACGAAT

***Miscellaneous***

180844+ 180862+ 18 degP GCGTTATCTGTTAAT

214202- 214227- -25 yaeP TTCTGCAATATCAGC

240301+ 240284+ -17 ykfE GCTGATATTGGAAAT

246255- 246261- -6 yafQ TTTATCAAATAAATC

246582- 246603- -21 dinJ TTTTACATAATTAAC

252211- 252222+ -11 yafO GCAAGATTAGAGGAA

265421+ 265366- -55 ykfB GGTATCAGTAGCTGC

294937- 294951- -14 yagN TATGGTAATACCTGC

311344+ 311328+ -16 ykgL ACAGTTTGAGTGAAA

398720- 398710+ 20 yaiZ TCACAAAATGGCAAC

398788+ 398782+ -16 yaiZ GCAGAATATGCAAGG

416223+ 416238- 15 sbcD TTTGCTTTTTTCTGC

453433- 453428- 15 yajG TTTGGTAAAAATTCC

455867+ 455875+ 8 clpP GCAGGTTTTTTCAAT

475536- 475550+ -14 ffs GCATAATCTGGAAAA

507321- 507324- 7 ybaP TGTGAAATATCATGC

515049- 515055- -6 ybbK TTAAACAAATTATAC

526625- 526689+ -64 ybbC GCGCTTAATAATAAA

527110- 527130+ -20 ylbH GAAATTAATAAGAAA

527752+ 527744+ -18 ybbD GAAGATAATAAAAAA

565455+ 565462- 7 ybcB CTTCACGCTTCATGC

570029+ 569993+ -46 ybcL GTATATTGTGGCAAA

579070+ 579095+ 25 ybcW GCATGGAGCGACAAA

592458- 592483- -25 nfrB TTTACCATTAATAGG

637783- 637813- -30 dsbG CTTTTCAGTCAGTGC

641190- 641229- -39 ybdQ TCTCCCAAAACCTGT

656393+ 656364+ -39 cspE GTAACTTCTTTAAAA

656436- 656422+ 24 cspE TCACTTTTTAGTAAA

657157+ 657140+ -27 ybeM ATAAAAACTGCTAAA

678687+ 678703- 16 ybeT GTAGTCAGTTAATGC

715994+ 715938- -66 ybfG ATTGCCACTTTTTTC

723712- 723728- -16 kdpD TTTGCGATCAGTTGC

807151- 807155- 6 ybhB GTTTGCAGAAAGTGT

823730+ 823730- -10 ybhP TTTTCCTGTCTTTGC

823814+ 823802- -22 ybhP TTTTCCGTAAAGTTC

830008+ 829991+ -27 rhlE ACAAAAACTGCTACA

832219- 832219+ 10 dinG CCAGAAACAGAAAAA

834404+ 834394+ -20 ybiB GAAGGTATAGTTAAA

840786+ 840776- -20 ybiL TTTCTTATATGTTGA

840887- 840885- 12 ybiL ATTATCACTTGGAGC

890084+ 890075+ -19 ybjC GCTATAACTGTTAAA

892042+ 891998+ -54 ybjN GAAGGAATAGAAAAA

892149+ 892132+ -27 ybjN GCGCTTTTTGCGTAA

906004- 906030- -26 ybjS TTTCCCCCATGATGC

932358+ 932323+ -45 ftsK GGAACAGGTGCAAAA

959343- 959337+ 16 ycaL GCAATAAATAGCCAA

969863+ 969820+ -53 ycaR GCACCGGCTGGGAAA

972721+ 972728- 7 ycbC TTTATCAGAAAACGT

1006936+ 1006937- -9 ycbX TTTGTCAGTTTGGCC

1027106+ 1027107- -9 yccT ATTCGTATTAGCTGC

1027150+ 1027145+ -15 yccU GTACACTTTGTTAAA

1034982+ 1034975+ -17 hyaD GCAAAATAAGTAACA

1034982+ 1034966+ -26 hyaD ACAAATTTGGCAAAA

1055404- 1055416+ -12 torT GCACTTTAGGTGAAA

1063250+ 1063211+ -49 yccE GCACTTTAAGTTTAA

1063250+ 1063234+ -26 yccE TCATTATTTATTAAA

1067123- 1067124+ 9 ymdF GCGGCTTATGGATAA

1084132+ 1084139+ 7 phoH GCAATAAATGCGAGA

1094979- 1094977+ 12 ycdU GCCCTATTTGGGGAA

1094979- 1094986+ -7 ycdU GGGGAATATGTTAAA

1094979- 1095047+ -68 ycdU GCATTTAGTGATGCA

1103986+ 1103999+ 13 csgC GCAAAAATTCTGAAA

1112662- 1112733+ -71 yceK GCCGCATCCGGCAAA

1124638+ 1124642- -6 yceL TTTATCAAAACGTCG

1125210- 1125281+ -71 yceH GAAGGCTATGCGAAA

1160851- 1160862- -11 fhuE TTTGTTTGTAAATGC

1164203+ 1164213+ 10 ycfP GCAGAACGTGTTACA

1166797- 1166792+ 15 ycfJ GCATGATTGGCATAA

1189051- 1189057- -6 phoQ TTGGGGATATTGTGC

1189169- 1189176- -7 phoQ CTTTGCCATTATTGC

1200268- 1200278- -10 ymfG TACTACACTAACTGC

1209456- 1209478+ -22 mcrA ACACTTTGTGTACAA

1209545+ 1209510+ -45 mcrA GCAACTTGTTGCAAT

1212359- 1212390- -31 ycgX TTTACCACTTCAATC

1221867- 1221883- -16 ymgD TTTACCCAGAATTGC

1257827- 1257826+ 11 ychH GCAGATAACGTAATA

1272899- 1272918- -19 ychN TTTTCCTGAAAGCGC

1272972+ 1272983- 11 ychN TTTTTCATTATTACC

1273089+ 1273100+ 11 ychO GCAAAAAGCGGCAAA

1276927- 1276964- -37 narX TTACTCCTTATTTGC

1288317+ 1288312+ -15 rssA GCAACATAGGCTATA

1292234- 1292283- -49 hns TTTCATAAAATTAGC

1297662+ 1297678+ 16 ychE GCATGTTTTGCACAA

1308920- 1308929+ -9 tonB GTACGTTATTTGAAA

1309081+ 1309024- -67 yciI TTTCACTGATCCTGA

1314116- 1314150- -34 yciG TTAGTCAAATTAAGC

1322637+ 1322662+ 25 yciQ GCAGAAACCGACAAC

1349814+ 1349815- -9 ycjD TTTGTCAGAATGAGG

1355725- 1355771- -46 ymjA TTTTATAGTTAATGG

1366062+ 1366049+ -23 pspA GCAAATTGTATTAAC

1389918- 1389913- 15 ycjY TTTAACGGTTGGTGA

1394109+ 1394110- -9 yghO TTTGTCATCTGGAGC

1403879- 1403911+ -32 ydaL GCAATATTTGGAACT

1411286+ 1411306- 20 intR TGTACCAGATAATCC

1418620- 1418632+ -12 ydaT GCAGAATATGTCAGT

1418681- 1418694+ -13 ydaT ACATCAAGAGGCAAA

1438931- 1438942+ -11 ydbJ GAATGTTTTGATCAA

1440965- 1440955+ 20 ydbH GGCGATATTGAAAAA

1473052+ 1473028+ -34 ydbD GCATTTACGGGAGAA

1488900+ 1488890+ -20 cybB GCCCGTTATGTTAAC

1488900+ 1488906+ 6 cybB GAAAATTATGTTAAA

1490398+ 1490391+ -17 trg GCAATAATTGTTGTA

1493157+ 1493168+ 11 ydcJ GCAAAATATTATTAA

1498507+ 1498511- -6 ydcK CGTAGCAAATTCTGC

1498554- 1498547- 17 ydcK AATTCCATAAAATGC

1500453+ 1500445+ -18 ydcL GCTATAACTTAGAAA

1500453+ 1500436+ -27 ydcL GCGCTTTTTGCTATA

1507112- 1507107- 15 yncJ TTTCTTACCTTTTGC

1507153+ 1507161- 8 yncJ TTTGCAAATTAGCGC

1521118- 1521147+ -29 yncE TAAATTTATGGAAAA

1521181- 1521169+ 22 yncE GCACAATACGTTAAG

1521181- 1521180+ 11 yncE TAAGTAATTGAGAAA

1521197+ 1521210+ 13 yncE GCAAGAAATGCTCCA

1521197+ 1521221+ 24 yncE TCCACATTTGAGAAA

1527915+ 1527916+ -9 ydcD GCAGATAATCCAATA

1528452- 1528445+ 17 yncI CCAGAAAGAGAAAAA

1590521+ 1590521- -10 hipB TTTATCCTTTAGTGA

1633917- 1633965- -48 ydfN TTTTCCAGTTCCGGA

1638808+ 1638789- -29 essQ TTTCATATTTAGTGT

1639746- 1639765- -19 cspB TTGAACATATGAAGC

1644332- 1644360+ -28 flxA ACAATTAATGACACA

1655523- 1655543- -20 ynfC TTACACGTTAAATGC

1669351+ 1669373+ 22 asr GGGTTAAATGAAAAA

1687781- 1687783+ 8 ydgA GAGCTTACTGAAAAA

1687880+ 1687863+ -27 ydgA TCATTTTATGGATAA

1702926+ 1702908+ -28 ydgT TCACTTTTGGTGAAA

1726343+ 1726327+ -26 rnt GTAATATTTGTCATA

1744159- 1744162- 7 ydhQ TTATTCCTTTGCTGC

1745115+ 1745125+ 10 ydhS ACACTAAATTCGAAA

1749983+ 1749915- -78 ydhW TTTGTCAGTACCTTC

1790169- 1790177- -8 ydiV TTTTTTAGATTATCC

1804194- 1804244- -50 ydiY TATTTCATTTTTTGA

1807374- 1807378- 6 yniB CCTTACACATAATGC

1830361+ 1830334+ -37 xthA GCATTTTTTGCGCCA

1846060- 1846104- -44 selD TGTCTGAATTCCTGC

1872329- 1872332+ 7 yeaL GCAGCAAGCGTAAAG

1891348+ 1891349- -9 yoaA TGTGGCAGTCTTTGC

1894061+ 1894076+ 15 yeaB CCATTAACGGACAAA

1894146+ 1894150+ -6 yeaB GAAACTTTTGATAAA

1894178+ 1894179+ -9 yeaB GAAGCAACTGGAGAA

1905612- 1905601- 21 cspC CTTTACTGAAAATGC

1905718- 1905732- -14 yobF TTCCACTTTTTTTGC

1905786- 1905792- -6 yobF CTTGCCAGTTCAGGC

1913045- 1913056- -11 yebJ TTTTACTGTTTTTGG

1913629- 1913632- 7 yobE GCTATCAGTAAATGC

1923425+ 1923445+ 20 yobB GCAGTCATTTTGAAA

1923956- 1924014+ -58 yobC GCACTATGGGATGAA

1928881+ 1928869- -22 yebG TTTCGTTTATACTGC

1944116- 1944105+ 21 yebB CGATGAACTGAAAAA

1944154- 1944157+ 7 yebB GCAAATATTTTAATA

1948762+ 1948769+ 7 yecD GCCCGAACTCATAAA

1957895- 1957890- 15 yecM TCTTTCAGTAAAAGC

1965496- 1965514- -18 cheY TTTGCCAACATTTGC

1985953- 1985966- -13 yecJ TTTTCAGGATTTTGC

1987667+ 1987654- -23 yecH TATGCCTTAACCTGC

1992900- 1992912- -12 uvrC ATCAGCATAATCTGC

1993792+ 1993763+ -39 yecF GCTATTTTTAGCAAA

2006231+ 2006220- -21 yedD TTTGTGAATTATTAC

2026448- 2026455- -7 yodC TTTTGCATAATAGCC

2075491- 2075493+ 8 yeeV GCCCGAAATGAAAAA

2145583- 2145607- -24 alkA TTATTCAGACGCTGC

2145671+ 2145653+ -28 yegD GCAACATTTCGCTAA

2165572- 2165570- 12 ogrK TTTTGTTGATGGTGC

2192291+ 2192275- -26 mrp TTTCCCATTTGGTAC

2209113+ 2209117+ -6 yehR GCAACAAGGATGAAA

2255339- 2255369- -30 yeiM ATTTTCCCTTCATGC

2263437+ 2263449- 12 yeiW TTTGACATTTTCGAC

2276301- 2276314- -13 yejG TTTAACACATCATCA

2276419- 2276436- -17 yejG TGTACCCGTTTCTGC

2282140+ 2282157- 17 yejK TATGCCACAAATTTC

2317890- 2317892+ 8 atoS GCAAGGTATTTCAAA

2411170- 2411189- -19 yfbV TTCAAAAATTTGTGC

2464537+ 2464531+ -16 intS GTAAAAAGTGGATAA

2493356- 2493391- -35 yfdY TCTGGCAATTTTTGC

2516283- 2516353+ -70 yfeC GAAAAATCAGGCAAA

2516851+ 2516833+ -18 yfeD GCAAATAATATGAAA

2549296- 2549289- 17 yfeY TTCACCAAAAAGTGC

2581577- 2581584- -7 yffH TTTGACTGATTTTAC

2614090+ 2614077+ -23 yfgC ACAGTATCGGTCAAA

2615571+ 2615590+ 19 yfgD GCATTAAAGGAAGAA

2628343+ 2628300+ -43 yfgI GCGTATTCAGCCAAA

2632138- 2632172+ -34 xseA CCCCTTTTTGCAAAA

2642401- 2642407- -6 yfgB TTGTACAAATTCTGG

2650473- 2650470- 13 yfhM TTTCACTAAACATCC

2672726- 2672742- -16 yphB TTAATCACAACTTTC

2685527- 2685543- -16 glnB TTTATAAAATTCTGT

2735110+ 2735086+ -34 yfiA ACAAAAAGTGACAAA

2739845+ 2739849+ -6 yfiR GAGCATTCTGAAAAA

2745981+ 2745990- 9 ffh TATGCCCGTTTTTGC

2763310- 2763314- 6 yfjL TTCCACAAATATTGT

2763343- 2763350- -7 yfjL TATATAACATACTGC

2763898+ 2763869+ -39 yfjN GCTAAATTTAGCAAA

2763911- 2763917- -6 yfjM TTCCCCATATGTTTC

2769107+ 2769120+ 13 yfjT GCTGATACCGTCAAA

2784699- 2784733+ -34 ygaR ACAGGATAGGAGAAA

2821841- 2821888- -47 recA TGTAGAAATTGTTGC

2821841- 2821885- -44 recA TTTTGTAGAAATTGT

2855046+ 2854979- -67 ygbA TTTCCCGGAACCAGC

2879094- 2879143- -49 ygcI TTGAGCAGTAATTGG

2879340+ 2879269- -71 ygcI TTTCGCTTTAACCGC

2928907+ 2928914+ 7 xni CCAAGTACCGCGAAA

2967516+ 2967533+ 17 mutH ACCAGATTTGGAAAA

2985430- 2985451+ -21 yqeH GCAAATACAGATTAA

2989165- 2989167+ 8 ygeG ACCAATAATGAAAAA

2991615+ 2991589+ -26 ygeI GCAAGATATTTAAAA

2996934- 2996974- -40 ygeQ TTTACAACAAAATGG

2998071+ 2998091- 20 ygeR TTTTACCCTATTTCC

3037798- 3037805- -7 xerD TATGCCTTATTGTGC

3037873- 3037899- -26 xerD TTTTACGGTTCCAGC

3052908+ 3052911- -7 pepP TTTTGTACTTCTGGC

3084085+ 3084050+ -35 yqgC GCTCCAACTGCGCAA=

3084107- 3084114+ -7 yqgC GAAAGTTCGGCGAAA

3093146+ 3093137- -19 yggR TTGCGCATAACCTGG

3144791+ 3144788- -13 yghW ATTGTCATATATAGC

3144955- 3144945- 20 yghW TTTGTCATAACGGGG

3147601+ 3147589- -22 yqhA TTTGTCGCATAATTC

3159280+ 3159299- 19 ygiQ TTGACCAACAGTTGC

3166410+ 3166405- -15 ygiT TTTTATAAAAGTCGC

3168485- 3168499+ -14 ygiY ACATTAGGTGAGAAA

3171102+ 3171116+ 14 ygiN GCATCTTGTGGAAAT

3171102+ 3171126+ 24 ygiN GAAATTTTTGGTTAA

3190060- 3190051- 19 glgS TTAAACATAACGTGC

3199053- 3199057- 6 ygiF TTTGTCAGTAGATAG

3199064+ 3199071+ 7 ygiM GTATTTTGCGCCAAA

3199064+ 3199075- 11 ygiF TTTGCGCCAAATTGC

3199110+ 3199133+ 23 ygiM GCAAGACCTGACACA

3232294- 3232303- -9 ygjM TTGAAAACATAATGC

3246345+ 3246335+ -20 yqjB GCCGGTATTTTTAAA

3296934- 3296954+ -20 yhbO CCCGATTGTGGGAAA

3351071- 3351066- 15 arcB TTCACGACAACCTGC

3359153+ 3359160+ 7 gltF GCTTTTTATTTTAAA

3364841+ 3364823+ -18 yhcF TCAAGAAGTGGTAAT

3375606+ 3375604- -12 sspA TTTACCAGATTTTGC

3395840- 3395835- 15 cafA TTTCTCACGCAGTGC

3409560+ 3409566+ 6 yhdJ TAAAAAATTGAAAAA

3430013- 3430057- -44 yrdD TCTTCCATTTGCTGG

3431607- 3431614- -7 smf CTTAGCAATCTTTGC

3464931- 3464929- 12 bfr TTTTTTAATTGCTGG

3465122+ 3465145- 23 bfd ATTGACAAAAAATGC

3510468- 3510467- 11 yhfZ TTTTCGGATTATTGC

3520848+ 3520857- 9 yrfD TTGTTTATAAACTGC

3530507- 3530529- -22 yhgE TTTTTCAGAAAATAA

3554889+ 3554881- -18 rtcA TTTATCCTTTTACGC

3582587+ 3582606+ 19 yrhB CGACGTTCTGAAAAA

3609782- 3609798- -16 yhhS TTTACCACGTGCTGA

3637902- 3637906- 6 uspB TTTTCCAGAAGGTTA

3654983+ 3654912- -81 hdeA TGCATCAGATACTGC

3656336+ 3656342+ 6 yhiE GTAAATAATGAAAAG

3694314- 3694309- 15 yhjR TTGCTCACTTTTTGA

3694314- 3694338+ -24 yhjS ACAGAAAAAGTTAAA

3694402- 3694404+ 8 yhjS GCTAAATCTGATAAG

3696175- 3696182+ -7 yhjU CCTCGTTATGTTAAA

3705783- 3705800- -17 dppA TTTTTTGTTTTTTGC

3705893- 3705879- 24 dppA TCCTCCATAAAATGC

3718009+ 3718016+ 7 cspA TCAAAATCTGTAAAG

3735238- 3735248- -10 bax TTTTTCACAAATTTG

3764250- 3764263+ -13 yibA GATATTAGTGCTAAA

3764250- 3764272+ -22 yibA GCTAAAGATGCAAAA

3765092+ 3765105+ 13 yibJ GAATTAGGTGATAAA

3766592+ 3766617+ 25 yibS GCTCAAAAAGCTAAA

3819409+ 3819357- -62 yicF TTGCGCAAAAAGTGT

3820057+ 3820023+ -44 rpoZ GCATGAGCCGCCAAA

3853641- 3853647- -6 yidF TCTCTCAATAAATAC

3865620+ 3865606+ -24 yidQ GCAGTTTATGGTCTA

3865620+ 3865602- -28 ibpA TTTTGCAGTTTATGG

3865631+ 3865641+ 10 yidQ GCACGAAACGTTAAA

3891791+ 3891785+ -16 yieE CAATAAAATGTAAAA

3957865+ 3957880- 15 ppiC TTTAGCATGATCCGC

3992265- 3992260- 15 cyaY TTTCCCTGTTTCTGG

4029040- 4029056+ -16 pepQ GTATTTTGTGTTAAA

4029152+ 4029137+ -25 pepQ GAACGATGTGCTAAG

4040165- 4040172- -7 mobA TGCTGCAATTTTTGC

4040165- 4040181- -16 mobA TTTTGCAGAAACTGG

4049256+ 4049236+ -30 yihI GCAAGGATTGTAAGA

4068562+ 4068548- -24 yihR TTTCGCATTAATATC

4135776+ 4135797- 21 yijF TTTCAGATTACTGGC

4175293+ 4175293+ -10 secE GCGTATAATCCGAAA

4228196- 4228223- -27 pepE TTAACCACTTACTGA

4255070+ 4255041+ -39 lexA CCAGAATTCGATAAA

4271956- 4271971- -15 uvrA TATGACACAAATTGA

4273192- 4273230- -38 yjcB TTAGCCATATTAGGC

4277939+ 4277934+ -15 yjcE CGATATTCTGAAAAA

4277939+ 4277943+ -6 yjcE GAAAAAAATGAGAAT

4324823- 4324833- -10 yjdM TTATACACAAAATCC

4366617+ 4366619+ -8 fxsA ACAATATAAGCTAAA

4370726+ 4370716+ -20 yjeI GCAGAAATAAACAAA

4398243+ 4398228+ -25 hfq GCTGAATGTGTACAA

4398258+ 4398259+ -9 hfq GCAATTTTTTCAGAA

4398296+ 4398298+ -8 hfq GCATATAAGGAAAAG

4437333- 4437343- -10 ytfJ TTTAATCATATGTGC

4532831+ 4532851+ 20 yjhR GCAGCATCTGGCAGC

4541009+ 4540968+ -51 fimA AAATAAACTGAAAAA

4542271- 4542305+ -34 fimC GCAGATAATGTGATA

4545612+ 4545636+ 24 fimF GCCTTTAGCGGGAAA

4573561+ 4573545+ -26 mcrD GCAACTATTGATATT

4584939+ 4584953- 14 hsdR TCTACCTGATTCTGC

***Metabolism***

122864- 122865+ 9 aceE GATTTTTCTGGTAAA

125650- 125658+ -8 aceF GCAGATAAAGTTAAC

141330- 141321- 19 gcd ATTAACAGATTCCGC

141362+ 141382- 20 gcd GGTGCCAAAACCTGC

148854- 148860- -6 panC TCAGCCATATACTGC

175012+ 175024- 12 hemL TTTGAAACATTACGC

175051+ 175051- -10 hemL TTTCATGGAACGTGC

188810+ 188806- -14 glnD ATGCTCATATTGTGC

229064+ 229071+ 7 yafB GCCAGAATCGCAAAA

234956- 234964- -8 gloB ATTGGCAATTTAAGC

243384- 243381- 13 yafH TTTAGCAATATGTTT

243384- 243385+ 9 lpcA GCAATATGTTTACAA

255771- 255773- 8 pepD GTTAGCAGAAAATGT

365567- 365573- -6 lacZ TTCCACACAACATAC

389003- 389018- -15 hemB TTTTACGAAAGTTGT

464796- 464791- 15 ybaX TTGAACAATAAAAGC

553218- 553213- 15 ybbF TTTGGCACGTCCTGG

557010- 557020- -10 folD TTTGTCAGAAGCGGG

712169+ 712162- -17 ybfF TTATTCATATACTCC

784771+ 784767+ -14 aroG GTTCATAGTGTAAAA

788233- 788250- -17 galM TTTGACGATTTCTAC

791418+ 791430- 12 galE TTTATGAATGATTGC

882655- 882667- -12 ybjG TTGAGAATAAAGTGC

882703- 882713- -10 ybjG TTTCCCTCAAGCGGC

952811- 952809- 12 pflB TTTTTTATTTACTGC

956792+ 956784+ -18 serC TCACTGAATGATAAA

960285- 960296+ -11 cmk GCTATAATTGCGCAA

982863- 982850+ 23 ycbL GTAGTAATTGCTTAA

2411339- 2411341+ 8 ackA GAAAATTACGCAAAA

4152910+ 4152917+ 7 argC GCAGTATTTATGAAT

3316514- 3316524+ -10 argG GCATTATTTTACAAA

3572960- 3572960- 10 asd CTTGGCACATCTTTC

3920627+ 3920628- -9 atpI TTTTCCAGTGCATAC

1955117+ 1955128- 11 bisZ TTTCGCTCATGCTGG

4173202+ 4173213- 11 coaA TGTGACAGATATTCC

4434704+ 4434704- -10 cpdB TATCACAGTTGGCGC

3989077+ 3989071+ -16 cyaA GCGAAAAGTGGTAAC

2597829+ 2597833- -6 dapA TATGGCATTAAAAGC

4254525- 4254548+ -23 dgkA GAAAGTCATGGGAAA

1151010+ 1151016+ 6 fabF GAAGAAGCTGAGAAA

2933571- 2933592+ -21 fucI GAATAAAGTGAGGAA

2936883- 2936903+ -20 fucU AGAGGAAGTGTGAAA

1686389+ 1686412- 23 fumA TCTCTCACTTACTGC

1686475- 1686499- -24 fumA TTTAACAAAAACTGA

1684674- 1684692- -18 fumC TTTACCATTTGATAA

1290493+ 1290511+ 18 galU GCATTACCTGCTAAT

1840332+ 1840330+ -12 gdhA TCGAAAACTGCAAAA

2507560- 2507584- -24 glk TTCAGCACCAATTGC

3559994+ 3559983- -21 glpE TTTGCTAATATGTTC

3352583+ 3352584+ -9 gltB GCAGTAATTGAGATC

2126352+ 2126362- 10 gmd TTTTTGAATTTATTC

3136559- 3136561- 8 gsp TTTAACAATACAACC

2632127- 2632122- 15 guaB GTTAATAAATATTGC

3331538- 3331551+ -13 ispB GAATATTACGCAAAA

1440907- 1440922- -15 ldhA TTTTACAAAATTTCA

3735492+ 3735487+ -15 malS GCAAACTGTCTGAAA

4118494- 4118500- -6 menA GCTCACATAATCTGC

2377397+ 2377385- -22 menD TTTGTAATAAAGTAC

2377397+ 2377417- 20 menD TTGTCGATTTCCTGC

4126556- 4126560+ 6 metB GCATATAATTTTAAC

865760+ 865758- -12 moeA TATAGCAAAAGTGGC

3772281- 3772303+ -22 mtlD TCACAATTTGTTGAA

2708360- 2708363+ 7 nadB GTAACAAATATTAAA

2643011+ 2642998- -23 ndk TTTAAAATAAAATGA

1531928- 1531927+ 11 nhoA GCAATAAGTAACGAA

2342780+ 2342781+ -9 nrdA GCAGTGAATCCCAAA

1980438- 1980450- -12 otsB TTGCGCAGAACGCGC

3530684+ 3530685+ -9 pck GCTATTTCTGCGATA

2699780+ 2699798- 18 pdxJ TTAGGCATAAACTGC

1990874- 1990880- -6 pgsA TTACCCCTATGTTGC

2966528+ 2966516- -22 ptsP TTTGGCGTATTTTCC

4402589+ 4402588+ -11 purA ACATGTTGAGGAAAA

4402688+ 4402703+ 15 purA GGTGATTTTGAAAAA

2595686+ 2595677- -19 purC TTTAAAATCTTTTGC

2693750+ 2693737- -23 purL GTTTACGCAAAATGC

1288021- 1288010- 21 purU TCTTTCAAAAATCGC

2110050- 2110058- -8 rfbD TTTCACATTATCAAC

3747997+ 3747990+ -17 sgbE GCATAAACTGAACTA

3820259+ 3820256+ -13 spoT GAAGAAAACGATAAA

2868486- 2868479- 17 surE TAATTCAGTTTCTGC

3667411+ 3667432+ 21 treF GAATGTTCTGAAAAT

1449524+ 1449542- 18 tynA ATGTACACATCATGC

4014411+ 4014423+ 12 udp GCTTCTTCTGACTAA

816024- 816039- -15 ybhK TTTCCCTTATTCTGT

1096902- 1096914+ -12 ycdW GCTAATTTAGGGAAA

1145848- 1145864- -16 yceF TTTTAAACTATGCGC

1405836- 1405842- -6 ydaM TTTATCAATTTTTGT

1438869- 1438876- -7 ydbK TCTTTCAATTTTAGC

1504782+ 1504793+ 11 ydcP TCAGCTACTGGATAA

1717805+ 1717811- 6 ydhH TTTGGTAGAACATGT

1860588+ 1860596- 8 yeaA TTTTATCGTAATTGC

1879901+ 1879925+ 24 yeaU TCAGGAAGTGAGAAC

1914271+ 1914278- 7 yebR TTTGGCACGTCATGG

1997532- 1997538- -6 yedO ATTTTCTTATTATGC

1997611+ 1997598- -23 yedO TTTTTGTCATTATGC

2241902+ 2241904+ -8 yeiG GAAGAAATTGCAAAC

2419545- 2419551+ -6 yfcH GCATGTAGAGAATAA

2696731+ 2696739- 8 yfhB TGTGTCTCATATTGC

2860220+ 2860212+ -18 ygbK GCCGATACAGCCAAA

2903464- 2903470- -6 ygcF ATTGTTAATTATTGC

2903464- 2903479- -15 ygcF TATTGCAGATCCTGC

3004296+ 3004284+ -22 ygeW GGACTTTATGATGAA

3202677+ 3202696+ 19 ygiH ACACGAATTTTCAAA

3217516+ 3217516+ -10 ygjG GCATATTTTGAACAG

3694022- 3694036- -14 yhjQ TTGCGCAAACTCTGC

4044649- 4044666- -17 yihG TTTAGCAGAAAACGA

4148361+ 4148371- 10 yijP ATTTGAATAAAATGC

4468950- 4468946- 14 yjgF TTTCTCCTTTATTAC

4593838- 4593860+ -22 yjjN GTAAATTTTGTGAAC

2815679+ 2815668- -21 yqaB TTTTGGAGAAGATGG

3176088+ 3176096- 8 yqiE TTTTACAGTTTGATC

2220063- 2220055+ 18 dld GAAAATGGTGCAAAA

2220190+ 2220200+ 10 dld ACAAGGAGTGGAAAA

4267378+ 4267391+ 13 aphA GCAACAAATCTCACA

1930849- 1930868- -19 eda TTTCACGCAAGGCGC

2363745- 2363757+ -12 yfbE GCAAAATGCCTCAAA

2363745- 2363768+ -23 yfbE CAAAATTTTGCCAAA

2363867+ 2363878+ 11 yfbE GCTCCAAATGGCAAA

2363867+ 2363888+ 21 yfbE GCAAAATATACACAA

3276888+ 3276885+ -13 agaZ GTATCAACTGAAACA

4254631+ 4254641+ 10 dgkA GCTCATAATGTAAAA

1104911+ 1104901+ -20 ymdB GCAGTATCTTGATAA

1108376- 1108397+ -21 mdoG GCACGGAAAGTGAAA

1078157+ 1078172- 15 putA GTTGCAACTTTGTGC

989648+ 989662- 14 pncB TTTCGCAATTTATTG

989738+ 989741- -7 pncB TGTAGCAATAAAGGC

606618- 606617- 11 ybdK TTTCTCTTATGTTGG

1839324- 1839331+ -7 ynjG CCCACTAATGTTAAA

4470711- 4470719- -8 pyrL GGTATCAATAATTGC

4606468+ 4606481+ 13 yjjG CGACGAACTGGAAAA

4631796- 4631807+ -11 gpmB GCAGTATACGGAAAA

1337214- 1337230- -16 ribA TTTGCCATAACGAGA

1337304+ 1337294- -20 ribA TCTGTCAATTTTTCC

1337181- 1337196- -15 ribA TTCTCCAGATAATGC

1337214- 1337220- -6 ribA ATTGGCTTATTTTGC

4141854- 4141853+ 11 pflD GCATCAAGTTTGAAG

4141938- 4141931+ 17 pflD CCATTATGAGCAAAA

1337214- 1337224+ -10 pgpB GCTTATTTTGCCATA

2576427- 2576418- 19 maeB TTTCACGTAACGTTC

2241821- 2241828+ -7 yeiG TCAGCAGATGTTAAA

3070677- 3070679- 8 pgk TTTTGCAGACGCTGC

4447681- 4447693- -12 ppa TTTAAAAATATGTGG

1732213- 1732221- -8 ydhD TTTGCTATTTTATCC

1653286+ 1653300- 14 rspA GTTGACAAAAGATGA

2654450+ 2654437- -23 pepB TTTTCCGCTAAATGA

2716697+ 2716699+ -8 trxC GTAACATATTAGAAA

3490397- 3490404- -7 ppiA TTTACCTAATTTTAC

3526640- 3526646+ -6 yrfG GCAGGTTATGCTACC

3643261- 3643258- 13 prlC CTTTACATAATGTGT

4014208+ 4014215- 7 ysgA TTATAAAAATGTTGC

3273229+ 3273247+ 18 yhaG TCATTTATTTTTAAA

3079739- 3079752- -13 tktA TTTACCGCAAGCCGC

4194859- 4194875+ -16 yjaD GTGAGAAATGTAAAA

1262216+ 1262220- -6 ychB CCTTCCAGTTTTTGC

1262216+ 1262229- 13 ychB TTTTGCCATTCTGGC

1635919+ 1635929- 10 ydfY TTTTACACAAACAGA

490530+ 490509+ -31 apt GCAATTATTGCGTAC

3984114- 3984156- -42 aslA TTATCCAGAAAATGA

808525- 808554+ -29 bioB ACACGTTTTGGAGAA

185985- 186025- -40 dapD TGATGCACTTCCTGC

4618812+ 4618796+ -26 deoD CCAGACTCTGGCAAA

2906147+ 2906094- -63 eno TTTCACAAAGCCTGC

2556797+ 2556743- -64 eutB TTTTTCACAGCATTC

2176653- 2176689- -36 fbaB TTTTCCATCTTTTGG

2261667- 2261703- -36 fruB TTTTGCCATTTTTCC

4137110- 4137152- -42 gldA TTGAGCATTTGTTGC

2350573+ 2350548+ -35 glpA ACAAAATGTTCAAAA

4390495+ 4390463+ -42 glyX CCAAAATTTGAAAAG

1363593+ 1363565+ -38 goaG TCAAAAGGTGTGAAA

499323+ 499306+ -27 gsk TCAGATTGTGTTAAC

2632138- 2632177- -39 guaB TTTTGCAAAAAATGC

2632138- 2632168- -30 guaB TCTACCCCTTTTTGC

4557669- 4557696- -27 iadA TTGCCCATTAAATAC

4557615- 4557644- -29 iadA TTTGGCATTCACTTC

2874392- 2874469+ -77 iap TAACGATTTGGCAAA

2347545- 2347594- -49 inaA TTTGCCACTACTAGC

4558916+ 4558889+ -37 kptA GCAGAAAATTCATAA

4163423+ 4163406+ -27 murI ACAGTTCGTGGTAAA

699578- 699608- -30 nagD TTTTCCAGCAAATGC

2342879+ 2342852+ -37 nrdA GAAACTATTGCGGAA

2342879+ 2342864+ -25 nrdA GAAAGAATTCCAAAA

149630- 149676- -46 panB TTTATCCGTAGCAGC

4105443+ 4105426+ -27 pfkA GCATTTTGTGTATAA

950287- 950319- -32 pflA TTCTCCAGATGTGGC

1991080+ 1991017- -73 pgsA TTCAACAACATTTGC

3482437+ 3482420+ -27 prkB GCACGAAACGGTCAA

1367686+ 1367656+ -40 pspE GCAAAAATTGTTAAA

4402683+ 4402657+ -36 purA GCTGTAACTCTGAAA

4402683+ 4402648+ -45 purA GCAAAAAGTGCTGTA

2595686+ 2595711- 25 purC TTTGTCGGATGCGGC

1287885- 1287921- -36 purU TTTTAAATTTTTTAC

2109124+ 2109063- -71 rfbA TTTAATTCATCTTGC

2110050- 2110105- -55 rfbD TTTTACGAATCCCGC

433835+ 433820+ -25 ribE AAATATTATGCTAAA

1292716+ 1292700+ -26 tdk ACGAATTATGATAAA

1246645- 1246671- -26 treA GATCGCACTAGCTGC

931330- 931360- -30 trxB GTTAACAATTTGTGC

3204301- 3204382+ -81 ttdA TCACGAACTGTTAAT

4014403+ 4014386+ -27 udp CGAAAAAATGTTAAA

2523087+ 2523028- -69 xapA TTTCTCTATTAATCC

27263+ 27247+ -26 yaaF CTAAGTTATGCGAAA

797778+ 797750+ -38 ybhE GCATTCACCGCCAAA

797689- 797717+ -28 ybhE CCACTATCGGACAAA

838418- 838443- -25 ybiX TTCTCCAAAAAGTGG

982721- 982750+ -29 ycbL GCAATATTCGCAAAG

1098080+ 1098044+ -46 ycdX GCGGCTACTAATAAA

1160964+ 1160948+ -26 ycfF GCAGAAATGGGAATA

1358991- 1359026- -35 ycjK TTTGTCAAATGTTAA

1374609- 1374676+ -67 ycjS GCATATTGCGGATAA

1732284- 1732310- -26 ydhD TTTAGCTTTCACTGC

2417319+ 2417296- -33 yfcD TTGATCATTGAGTGC

2458531- 2458557- -26 yfcY TTTTTTACATTTAGC

2998277- 2998305+ -28 ygeS GAACCAAATGTTCAA

3005375- 3005445+ -70 ygeX GCAACTGCTGAAAGA

3319803+ 3319755- -58 yhbX CTTTTTAAATGATGC

3788312+ 3788289- -33 yibD TTTTACATATTTAAC

4071737+ 4071714- -33 yihU TTAAACAATTGGTGA

4414946+ 4414929+ -27 yjfP GAAACTTGTGCTAAG

694243- 694273+ -30 yleB GCCCTTAGGGGTAAA

2550192- 2550223- -31 ypeA TTAGCCAATATCTGA

3154496+ 3154454+ -52 yqhE GAAAAAACTGGAAGA

3176084+ 3176060- -34 yqiE TTTTGCGGTAGCGGC

541120+ 541092+ -38 ybbZ GAATAATTTGTTGAA

1402725+ 1402653- -82 abgA CTTATCACTAACTGA

1402725+ 1402670- -65 abgA TTTCACAGTTTTAAC

4492508- 4492539- -31 idnD TTACGCATAACGTGA

557010- 557053- -43 folD TTTCAGATTTATCGC

3968009+ 3967993+ -26 rffE GGAAGAACCGGTAAA

9131- 9162+ -31 mog GGAAAAACTGGAAAA

3760168+ 3760106- -72 yibF TTTCCTAATTATTTC

1193481- 1193532- -51 ymfB TCTGTCACTTCTCGC

***cyclic-di-GMP signalling***

872121+ 872107+ -24 yliE GCTGATTTTGTAGAA

872123+ 872145+ 22 yliE GCAGCAAGGGACAAT

1896252+ 1896272+ 20 yoaD CCAAATACCGCGAAA

1609965+ 1609977- 12 yneF TTTGTCGTTTTCCGC

2515989- 2516007- -18 yfeA TATTACAAAAGCGGC

2141147- 2141155+ -8 yegE GTAAAAAATGTTAAA

2141228+ 2141219+ -19 yegE ATATAAAATGTAAAA

2141233- 2141245+ -12 yegE GCATAAACACAGAAA

2026068- 2026080- -12 yedQ TCTGGCATAACCTGG

2026218+ 2026217- -11 yedQ TTTCACACTTCTTCC

402904- 402908+ 6 yaiC CTATAATTTGGGAAA

1621901- 1621893- 18 ydeH TTTCACAGTTCCTTG

1405893- 1405920- -27 ydaM TTTAACACTTCACAC

2740257+ 2740233+ -34 yfiN ACAATATCCGTCAAA

2141228+ 2141199+ -39 yegE TCACTTTATGAGCAA

***Electron transport, oxidative stress, anaerobic respiration***

1480928- 1480936- -8 acpD GTTGACACTTTATTC

638104+ 638117+ 13 ahpC GCCGAATCGGCAAAA

638132+ 638126+ -16 ahpC GCAAAAATTGGTTAC

4457484- 4457493+ -9 cybC GGATGAAGTGTAAAT

2297709+ 2297710- -9 napH TTTCGCACTTACCGC

1540709+ 1540708- -11 narZ TTTGGCTGAACTTCC

1067044- 1067044- 10 wrbA TTTATAACAACTTTC

1067084+ 1067072- -22 wrbA TTTGTCGCAAAATGA

1778398- 1778385+ 23 ydiR GGAACATCTGAAGAA

6481- 6497- -16 yaaA TTTAGCAAAAAATGA

3028988- 3028990- 8 ygfT CTTCTCAAATTTCGC

3170452+ 3170463+ 11 mdaB GCTAAATTCGCAAAA

3236417+ 3236439+ 22 ygjT TTACGATGTGTGAAA

1846762+ 1846774- 12 ydjA GTCCGCAATTCCTGC

1846857+ 1846879- 22 ydjA TTGGCGATTTATTGC

2798622- 2798625+ 7 nrdH CCACTATTTGCTATA

2225316- 2225304- 22 yohF TTCTCCACTTAACGC

2898384- 2898370- 24 ygcW TTTCTCATTATCTCT

518388+ 518383- -15 ybbO TTTACTAAAGGCTGC

1722703- 1722752- -49 sodC TATTCCTAAAAGTGC

1488900+ 1488874+ -36 cybB TGAAATTTTGAGAAA

450931+ 450956- 25 cyoA GGTCACATATGTTGC

2293118+ 2293060- -68 ccmF TTTTCCAGTTCGCCC

4079928+ 4079903- -35 fdoI TCTTTCTCTTCGTGC

1684674- 1684713- -39 fumC TTTGGTCTTTCGTGC

1723834+ 1723770- -74 ydhF TTGCGCATTAATCGC

943153- 943180+ -27 dmsC GAATATTGTGATCAA

2403222- 2403266- -44 nuoA TTTAACAAAAGTTTC

3170500+ 3170484+ -26 mdaB GCACATTTTGCTAAT

1655894+ 1655919+ 25 ynfE GCCGGATATGACAAA

1658506- 1658570+ -64 ynfF GCAATAAGTGAGTAA

1986706+ 1986680+ -36 ftn GCAAATAATAAAATA

4287703- 4287731+ -28 nrfC ACGTTAAGTGACAAA

1863678- 1863725- -47 yeaE TTATGCACAAAATGC

2037435+ 2037402+ -43 yedY GCATAAATATGTAAA

2037435+ 2037393+ -52 yedY GCAGTTAATGCATAA

2668443+ 2668404+ -49 yfhV GCAGGAGGTGATGAA

^1^ the + or – suffix denotes orientation of the transcription start site (TSS)

^2^ the position is the centre of the marbox, the + or – suffix denotes orientation

^3^ sequence in the 5' to 3' direction on the top DNA stand
