# Supplementary material for: Coordination of cell envelope biology by Escherichia coli MarA protein potentiates intrinsic antibiotic resistance
Source: PLoS Genet. 2025 May 5;21(5):e1011639. doi: 10.1371/journal.pgen.1011639 (PMC12052159; doi:10.1371/journal.pgen.1011639)
Supplement: S3 Table — (DOCX) [file pgen.1011639.s009.docx]

**Table S3: Identification of known MarA target genes**

**Known MarA target gene^1^ Identified by this work Identified by ChIP-seq^2^**

*lacZ* Yes N/A

*acrR* <> *acrA* No No

*nfsB* No No

*ybjC* <> *grxA* Yes Yes

*pqiA* No No

*ycgZ* <> *bluF* No N/A

*acnA* No No

*marR* <> *marC* Yes Yes

*fumC* Yes No

*zwf* <> *yebK* Yes No

*micF* <> *ompC* Yes Yes

*inaA* Yes No

*xseA* <> *guaB* Yes N/A

*tolC* <> *nudF* Yes Yes

*mlaF* <> *yrbG* Yes N/A

*hdeA* <> *yhiA* Yes No

*rfaY* Yes No

*sodA* <> *rhaT* No No

*fpr* No No

*purA* Yes No

*rob* <> *creA* Yes No

^1^Sites listed by the Ecocyc database as supported by high quality evidence were used^1^. The exception is *tolC*, where high quality evidence has been published but has not yet been added to Ecocyc^2^. Note that *mlaF* is also referred to as *yrbF*. Sites between divergent genes are denoted by “<>”.

^2^Sites first discovered by our prior ChIP-seq^3^ and subsequently added to Ecocyc are listed as “N/A”, since these were not known MarA targets at the time the ChIP-seq was done.

**References**

1. Keseler IM, Mackie A, Santos-Zavaleta A, Billington R, Bonavides-Martínez C, Caspi R, Fulcher C, Gama-Castro S, Kothari A, Krummenacker M, et al. 2017. The EcoCyc database: reflecting new knowledge about *Escherichia coli* K-12. *Nucleic Acids Res* 45:D543–D550.

2. Zhang A, Rosner JL, Martin RG. 2008. Transcriptional activation by MarA, SoxS and Rob of two tolC promoters using one binding site: A complex promoter configuration for tolC in Escherichia coli. *Mol Microbiol* 69:1450–1455.

3. Sharma P, Haycocks JRJ, Middlemiss AD, Kettles RA, Sellars LE, Ricci V, Piddock LJ V, Grainger DC. 2017. The multiple antibiotic resistance operon of enteric bacteria controls DNA repair and outer membrane integrity. *Nat Commun* 8:1444.
