# Supplementary material for: Coordination of cell envelope biology by Escherichia coli MarA protein potentiates intrinsic antibiotic resistance
Source: PLoS Genet. 2025 May 5;21(5):e1011639. doi: 10.1371/journal.pgen.1011639 (PMC12052159; doi:10.1371/journal.pgen.1011639)
Supplement: Attachment — Submitted filename: Response to reviewers.docx [file pgen.1011639.s012.docx]

We thank all of the reviewers for their supportive comments. We have made all of the revisions requested or have explained why we think the changes are unnecessary (a small number of differing opinions on presentational style). In a couple of places, the reviewers asked for statistical tests. For completeness, we have added these throughout.

Reviewer's Responses to Questions

**Comments to the Authors:
Please note here if the review is uploaded as an attachment.**

Reviewer #1: The manuscript submitted by Trigg et al. describes a detailed analysis of selected members of the MarA regulon in E. coli and how they synergistically play a role in contributing to intrinsic antimicrobial resistance. The study is elegant and thorough, combining clever bioinformatic identification of likely MarA binding sites followed up with convincing molecular studies to verify the targets. I have no major concerns, but highlight several points below that, if clarified, would improve the quality of this manuscript:

• The background information cited on MarRA, SoxS and Rob can be confusing in parts. As these factors and their regulated genes are intrinsic, what are the natural signals for activating MarA (or even SoxS/Rob) regulation or MarR derepression? Salicylic acid is mentioned but how is this relevant? Is MarR derepression or activation of MarA regulated genes ever triggered by exposure to drugs? Can you elaborate on what stressful conditions (line 447) might be in reality? This would be useful for the reader to contextualise the otherwise beautifully presented data.

This information (now expanded to better explain the system) is included in the intro (starting line 56). The *marRAB* operon is induced when MarR releases the DNA. This can be due to phenolic compounds, copper regulated disulphide bond formation between MarR molecules, and mutation of marR or its DNA site. MarA cannot sense signals directly, so is controlled only at the level of its expression, via MarR. We have also added more information for the other regulators (SoxS and Rob) later in the introduction. On line 447, we now make no reference to “stressful conditions”. Instead, we talk about loss of repression by MarR and refer the reader back to text in the introduction.

• The bioinformatic pipeline is clever but somewhat arbitrary. I agree that their choice of targets to follow up based on Figure S1 was logical but is there a possibility that interesting MarA targets may be missed? Is there any published transcriptome data of marA mutant cells that could be integrated here to assess other potentially regulated genes?

Most likely we will have missed some targets, but that is always the case with large scale approaches. However, the number of false negatives is much lower than our prior ChIP-seq analysis, which identified only 24 % of known MarA targets. Our current analysis identified 70 % of known targets (see the new Table S3). We have now added this information to the end of the intro and in the appropriate results section. Transcriptomic data do exist but they are notoriously difficult to interpret for MarA because i) SoxS and Rob can step in when the former is deleted and ii) there are many pleiotropic effects. Furthermore, much of the existing transcriptomic data are contradictory. The best analysis of all existing transcriptomic data, highlighting the problems, is in PMID: 12067348.

• How do the authors reconcile the lack of in vitro effect in figure 5c? I was not clear on what they concluded was actually going on here. Is it possible that both MarA and SoxS are needed in tandem? If they are added in combination to the assay in figure 5d do you get an exaggerated effect?

We have seen, in prior work, that the preference of a given site for binding MarA or SoxS can switch in response to buffer conditions *in vitro*. This is related to the different ways in which MarA and SoxS recognise their target. It may be that, in our in vitro assays, binding of SoxS is favoured. Conversely, *in vivo*, both regulators exert a similar effect, suggesting both can bind. Note however that this binding could not be simultaneous, since MarA and SoxS recognise the same DNA site. We have added this explanation to the appropriate results section.

• Effect of pbpG in Figure S3 is difficult to discern. Are these statistically significant changes?

Yes, this is significant, and statistical tests have been added both to this figure and throughout the text.

• Figure 6a – it would be helpful if you provide a key for the colour scheme on the heatmap

Apologies for this omission. This has been corrected.

• Line 382 – Should be spelled “murein”

This sentence has now been altered to address the comment below (better explaining synergy). We now refer to peptidoglycan.

• Mechanistically how does PbpG and LpxC synergy work? I am not clear on what the authors are proposing here? Are they suggesting that these independent systems could somehow interact or that the synergy is purely at the regulatory level? It seems counterintuitive that its not a cumulative fitness defect and that they are entirely co-dependent? Perhaps I am missing something here that could be clarified in the discussion.

We do not see synergy between *pbpG* and *lpxC* regulation (if this is what the reviewer means). Rather we see synergy between each of these regulatory events and control of the *mlaFEDCB* operon. All three regulatory events are likely to improve barrier function. For PbpG, repression will cause reduced crosslinking between cell wall glycan strands and so reduced cell wall porosity. For LpxC, activation will increase LPS production, improving outer membrane barrier function. Similarly, activation of *mlaFEDCB* will remove phospholipids from the outer leaflet of the outer membrane, again making a better barrier. Re synergy, it seems likely that loss of one aspect of the barrier function is less of an issue, because the others compensates. Loss of two barrier components could have a much bigger effect that is more difficult to compensate for. We have updated the first paragraph of the discussion, and the penultimate results section, to better explain this.

• Figure 7a – I don’t think the cell schematic is necessary. The images of the mutations is sufficient.

We’d like to keep this to make it clear that the changes are simultaneous and on the chromosome.

• Figure 7c – can you calculate some statistical analyses on the growth rates to quantify the extent of the fitness defects?

Yes, this has been added (see text and new Figure S5).

• Line 405-406 - What could the compensatory factors be? SoxS or Rob? Have you tried these assays in Rob/SoxS mutant backgrounds?

No, we do not mean compensation by SoxS or Rob. We mean factors that might compensate for the loss of *pbpG*, *rfaY* and *mlaFEDCB* expression. E.g. cell envelope barrier function could be altered by expression of other proteins involved in cell envelope biology. Alternatively, unrelated transcription factors could intervene to control *pbpG*, *rfaY* and *mlaFEDCB* (it seems likely that MarA/SoxS and Rob are the only regulators for these genes). We do not think doing the assays in a background lacking Rob/SoxS would be worthwhile for two reasons. First, we have mutated the DNA site shared by MarA, SoxS and Rob. Hence, any regulatory effect of the 3 factors will have already been removed. Second, doing experiments in cells lacking SoxS or Rob would induce many changes in gene expression, unrelated to *pbpG*, *rfaY* and *mlaFEDCB*. Hence, alterations in MIC etc would be impossible to interpret. Indeed, this is why we instead opted to mutate specific binding sites, rather than delete the regulators completely. Whilst editing the paper, we reasoned that altering all three marboxes could lead to suppressor mutations elsewhere in the genome, so have also included this caveat in the revised text.

• Line 467 – Should be spelled “oxidative”

Corrected

Reviewer #2: The manuscript of Trigg et al., describes a study in to the multi-antibiotic resistance activator proteins MarA that has been implicated in intrinsic resistance to antibiotic in enteric bacteria. The authors used a computational screen to determine the functional MarA binding sites based on proximity to know transcriptional start sites in the E coli genome. The authors then used genetic and biochemistry to interrogate the activation of potential targets such as genes affecting LPS biogenesis and PG remodelling. Uncoupling of both these processes from MarA regulation results in increased sensitivity to envelope stress and makes cells hypersensitive to mutations in the lipid membrane shuttling system, Mla that transports lipids between the two membranes in didermic bacteria.

The work is well conducted and makes a novel contribution to our understanding not only of intrinsic resistance but also in terms of interplay between envelope remodelling and antibiotics. This is particularly important given that MarA overexpression can allow acrAB and tolC mutants to become resistant to certain antibiotics and yet understanding this mechanism has been elusive to date due to the difficulty in identifying bona fide MarA binding sites.

I think the work is suitable for publication as it is, but i have a few minor points for the authors to consider

I wonder if the authors saw any MarA binding upstream of the Mce domain containing proteins that potentially have similar function to MlaD such as PqiAB or YebST? and or doe these mutations give the same phenotype?

Yes, there is evidence for this. First, MarA is a known regulator of *pqiAB* (e.g. PMID: 21097628). Second, our bioinformatic analysis identifies a potential marbox upstream of *yebS*, although we have not confirmed MarA binding by gel shift assay. Mutation of *pqiA* gives a similar phenotype to mutation of *mlaE* with respect to tetracycline resistance . Neither *yebS* or *pqiA* mutation has much impact on doxycycline or minocycline resistance.

Figure 7 B & C - growth curves should be on a log scale or semi-log plots

This is a reasonable suggestion but, we think, also a matter of personal choice. Using a linear plot does not obscure the result and this is the way we present growth curves in all of our papers (e.g. PMID 29133912). Since altering the graphs makes no difference to the data or interpretation, we prefer to use our usual style.

Figure S5 amiC and wbbK are not very convincing shifts...

Apologies for any confusion. We agree that MarA does not bind these targets and this was our conclusion in the paper. We suspect the reviewer is confused because the whole gel has run at an unusual angle (a slant from left to right). The bands all correspond to unbound DNA. For clarity, the below are the relevant excerpts from the paper.

“The results of the analysis are shown in Figure 1c. Whilst MarA did not bind to any of the control DNA fragments all but one (amiC) of the predicted targets was bound by MarA in vitro.”

“Next, to determine if MarA was able to regulate transcription from the targets identified, the various DNA fragments were cloned upstream of lacZ in plasmid pRW50. As a control, we also cloned three of the promoter regions that did not bind MarA (amicC, waaD and wbbK)”

Reviewer #3: The study has an exciting goal of using a combination of computational and experimental techniques to identify novel MarA binding sites. It provides an interesting computational approach and succeeds in finding novel MarA binding sites for several genes related to LPS and cell wall remodeling using a variety of experimental techniques.

Since the computational approach is a basis for the detailed experimental investigations, I wish it is described and evaluated in more details. Why were the sequences of the predicted sites aligned to the MG1655 genome when the study is done with JCB387?

MG1655 and JCB387 are extremely similar. Both strains are E. coli K-12, so have the same genome sequence. Only the genotypes are slightly different, with JCB387 being lac-, and so suitable for LacZ assays. All of the predicted sites occur in both strains.

Do the targets identified with this approach overlap with the ones identified by other approaches?

Yes, there are many overlapping sites but our new approach presented in this paper is much more powerful, identifying 70 % of known targets. Our prior ChIP-seq found only 20 % of known targets. These details are now added in the introductory and results sections.

How were the targets for further investigations selected? Did they stand out statistically or they are chosen completely randomly?

There were three steps to the selection. First, we identified gene functional groups that were overrepresented adjacent to marboxes (Figures 1b and S1). Second, for each gene group, we selected several sites to study, some involved in cell wall biology and some in LPS production (this step was arbitrary). Third, the experimentally tested for binding of, and regulation by, MarA (Figures 1c and 1d). Where both activities were apparent, the targets were followed up further.

The experimental results for identifying the marboxes and their classes are interesting, but the figures could be improved to enhance the clarity. Many figures could benefit from better annotations in figure instead of putting explanations in the figure legend.

We have made alterations throughout the figures to better annotate gel shift assays and diagrams.

The gel images should be clearly labeled with DNA/protein size bars on the side

Gel shift assays require no size marker since only a single labelled DNA, of known size, is run on the gel. This is consistent throughout the literature and in PLoS Genetics.

The primer extension assay does require a marker and this is shown (Figure 2e).

For *in vitro* transcription assays, an internal size standard (the 108 nucleotide RNAI) is produced by the reaction and is a useful size comparison for transcripts from cloned promoters, that are slightly larger RNAs. Note that no other RNAs of a similar size are present, so there is no potential for identifying bands incorrectly. Additionally, we can see that our transcripts of interest are subject to regulation by mutation and/or the presence of MarA/SoxS, again confirming that they are the correct bands. Lastly, for all *in vitro* transcription DNA templates, we ran at least one gel with size standards (see figure S6) to confirm the transcript of interest was the size expected.

There are no gels where protein size markers are relevant; all gels show DNA or RNA.

and the heatmaps need to have a colorbar legend to indicate the values the colors represent.

This has been added

Also, there are several places in the text where the readers were directed to compare parts of a specific panel to another part from a panel from a different figure. The writers should consider organizing the figures better so it is easier for the readers to understand the results.

This is not true, we ask for several comparisons within or between panels (usually comparing lanes within the same gel image) in the same figure. Only once do we ask reviews to compare between figures (Figures 3a and 4a, to highlight the different promoter organisations). This does not seem unreasonable.

There are 12 subsections of the results section. Each section is short and discusses different things.

We are not sure if this is a criticism or just a statement. Clearly, the sections have to discuss different topics, and they do not seem unusually short.

While they are rife with information about experimental design and presentation of the results, they do not offer detailed biological interpretation of the results.

We prefer to keep detailed interpretation for the conclusions section, although some is present already in the results to help move the story along. Again, we note that the other reviewers commented on the clarity of the presentation being good.

Any theories on why the in vitro assay did not show the influence of MarA on the expression of pbpG while the in vivo assay did?

We have seen, in prior work, that the preference of a given site for binding MarA or SoxS can switch in response to buffer conditions *in vitro*. This is related to the different ways in which MarA and SoxS recognise their target. It may be that, in our in vitro assays, binding of SoxS is favoured. Conversely, *in vivo*, both regulators exert a similar effect, suggesting both can bind. Note however that this binding could not be simultaneous, since MarA and SoxS recognise the same DNA site. We have added this explanation to the appropriate results section.

Any hypothesis on why cells lacking *pbpG* have increased fitness when exposed to tetracyclines, a drug that interferes primarily with protein synthesis, but show mixed patterns for drugs affecting cell envelope, which *pbpG* is directly associated with?

The impact of deleting *pbpG* is increase cell wall glycan cross linking, and so barrier function. Hence it is more difficult for tetracyclines to diffuse across the cell envelope and find their target. This is expanded upon in the revised text. Conversely, any molecules that don’t need to traverse the cell wall, or do so regardless of PbpG activity, are unlikely to be impacted by *pbpG* deletion, regardless of their protein target.

The connections between some of the 12 sub-sections and how they contribute to the overall conclusion of the study is not obvious.

Without a more specific comment we do not known which sections the reviewer means. We have read through the text and, where we think appropriate, have made some small modifications to better link one section to the text. Overall, we felt that the text read well already and that the links are obvious.

The results appear scattered instead of coming together as a holistic and convincing story. It would be great to see some of these results reorganized and condense to have a simpler structure with more detailed biological discussion of what the results actually mean in the biological context.

Again, without a more specific suggestion, it is difficult to respond. In our view, and the view of the other reviewers, the story is well presented and very convincing. Hopefully, the various changes we have made address this concern.
